# Smiling in time: the interactive development of infant smiling with mothers, fathers and strangers

**DOI:** 10.1098/rstb.2025.0142

**Published:** 2026-09-24

**Authors:** Eliala Alice Salvadori, Daniel S. Messinger, Alan B. Cobo-Lewis, Itir Onal Ertugrul, Cristina Colonnesi

**Affiliations:** ^1^ Clinical Child and Family Studies Unit, Research Institute of Child Development and Education, Faculty of Social and Behavioural Sciences, University of Amsterdam, Amsterdam, The Netherlands; ^2^ Department of Pedagogy, Psychology and Philosophy, University of Cagliari, Cagliari, Italy; ^3^ Department of Psychology, University of Miami, Coral Gables, FL, USA; ^4^ Departments of Pediatrics, Music Engineering, Electrical Computer Engineering, University of Miami, Coral Gables, FL, USA; ^5^ Emergency Smile Research Project, RED NOSES International; ^6^ Department of Psychology and Center for Community Inclusion and Disability Studies, University of Maine, Orono, ME, USA; ^7^ Social and Affective Computing Group, Department of Information and Computing Sciences, Utrecht University, Utrecht, The Netherlands

**Keywords:** infant smiling, positive affect, interaction, scaffolding, survival analysis, development, emotional development

## Abstract

A developmental examination of the temporal dynamics of infant smiling during early social interaction is lacking. We ask how infants and adults influence each other's expressions of positive affect by examining the timing of transitions among dyadic states defined by the presence and absence of each partner's smiles. Fifty-eight infants interacted with their mothers, fathers and a stranger at 4 and 8 months. A survival analysis of the time course of infant (and adult partner) smiles and non-smiles indicated that infants initiated smiles and reciprocated smiles at lower rates than their adult partners, yet ended smiles at higher rates. Nevertheless, adult smiles were associated with higher infant smile rates, while infant smiles prolonged adult smiles, suggesting a dyadic path to mutual positivity. Eight-month-olds initiated smiles at higher rates than 4-month-olds when neither partner was smiling, suggesting increasing infant smiling initiative. Partner-specific patterns revealed selective infant responsiveness. Although strangers initiated and sustained smiles at higher rates than mothers, infants were more probably to reciprocate mother than stranger smiles. In the first year of life, infants showed a tendency to down-regulate positive affect expressions across age and context, as well as partner-specific smile reciprocation proclivities and a developmental increase in smile initiation.

This article is part of the theme issue ‘Mechanisms, development, phylogeny and functions of emotional expressions’.

## 1. Introduction

The early expression of positive affect is fundamentally social. Despite widespread recognition that social interaction provides a medium for affective development, we know surprisingly little about the temporal organization of infant smiling during interactions with social partners. How does the presence of an adult smile influence the rate at which infants initiate, reciprocate and end smiles? Do these dynamics change across early development and vary depending on the adult partner with whom the infant is interacting? These questions are at the heart of theories emphasizing the interactive construction of emotion [1,2]. Yet, existing research rarely focuses on the temporally dependent nature of positive affective expressions and their development. For example, little is known about how smile-based turn-taking develops over the first year of life, how infants and adults coordinate pathways to mutual smiling associated with shared joy, or how the development of smile initiations may support infants' later referential communication of positive affect. Understanding the temporal dynamics of infant smiling across partners is essential for characterizing how positive affect expressions emerge and support early regulation and social development. Here, we model how the presence of adult partner smiling influences the rate of infant smile onsets and offsets between 4 and 8 months during infant face-to-face interactions with mothers, fathers and strangers.

### (a) Infant smiling over development and across adult partners

Infant smiling, an index of positive affect, emerges within and contributes to social interaction [1,3]. Although early smiles in the first weeks of life occur predominantly during sleep and periods of drowsiness, by 6–8 weeks, infants begin producing smiles in response to social stimuli, particularly faces and voices [4–9]. Social smiles increase in frequency across the first year and become increasingly socially directed [3,10,11]. By 4 months, infants smile more reliably during face-to-face interaction, and these smiles serve communicative functions—eliciting caregiver responsiveness, maintaining interaction and signalling positive engagement [5,12–15]. Finally, infant mother smile-based turn-taking increases over the first 6 months, suggesting that infants and mothers become more responsive to both the onsets and offsets of one another's smiles [13]. While the developmental trajectory of smile *frequency* and *duration* is well-documented, we know less about the temporal organization of infant interactive smiling—how real-time production of infant smiles changes across development and interaction partners.

The period from 4 to 8 months is marked by noteworthy developments in social cognition and interactive capacity. During this period, infants increasingly coordinate attention between objects and people [14], show emerging awareness of others as intentional agents [16] and display more sustained and reciprocal interaction patterns [17]. Between 3 and 6 months, infant temporal coordination of their own smiling and gazing at the mother's face increases [11]. At the same time, infants' more affectively positive smiles become increasingly coordinated with infant gazes at mother's face and with mother smiling [18]. Likewise, infant initiation of smiles to familiar and unfamiliar adults at 8 months appears to play a role in the referential communication of positive affect [19–21].

While researchers have focused on infant positive affect during infant–mother interaction, infant affective dynamics differ by partner. Mothers and fathers display distinct interaction styles: maternal interactions tend to be characterized by nurturing, soothing and protective behaviour, while paternal styles tend to be more dynamic, playful and stimulating [22–24]. These stylistic differences are reflected in affective displays, with mothers engaging in longer expressions of positive affect than fathers in infant interactions [25–27]. Correspondingly, infants typically smile longer with mothers than with fathers during face-to-face interaction (e.g. [26,28,29]), and longer with mothers than with strangers [29–31]. Together, these findings indicate that infants modulate their early expressions of positive affect based on partner identity and relationship history. Yet, it is not clear whether partner-specific differences in levels of positive affect reflect variation in the temporal organization of infant smile production.

### (b) Theory and analytic approaches

Dynamic systems, mutual regulation and other theoretical perspectives hold that infant positive affect is co-constructed by both infant and adult behaviour in real time, with each partner's actions continuously influencing the other's affective trajectory [25,32,33]. This suggests that infant smiles are not best understood as isolated expressions but emerge through a dynamic feedback loop in which each partner's behaviour influences, and is influenced by, the other [3]. While researchers typically document the frequency and duration of infant smiles and those of their adult interaction partners (e.g. [18,28,29,34]), understanding coordination requires a complementary focus on the temporal dynamics of infant and partner smile onsets and offsets.

Several approaches have proved fruitful in understanding early infant–parent smile dynamics. Sequential analyses indicate that infant smile onsets typically elicit mother smile onsets within a 1–2-second window [35]. However, while the presence of mother smiling is all but necessary for the occurrence of infant smile onsets, infants typically do not begin a smile within a second of mother smile onsets [36]. Sequential analyses often involve calculation of likelihood that infants begin a smile after an adult smile onset while accounting for the base rates of both partners' smile onsets, providing evidence for interactive contingency [37–39]. However, contingency analysis typically requires making *a priori* or data-informed decisions about the fixed intervals over which contingencies will be tallied. More fundamentally, contingency analyses do not account for the partner's continuing action. This is important because the continuation of an infant smile after its onset typically depends on the adult partner continuing to smile [36]. By contrast, time-series analyses focus on the ongoing affective behaviours of both infants and their partners. However, time-series approaches typically combine multiple behaviours into ordinal affective spectra (e.g. negative to positive [40]). As a result, while such models can detect cross-partner influence, they typically cannot determine how specific infant and mother smiling behaviours influence the other partner [40,41].

The current article asks how the smiling actions of infants and adults create a dyadic context that influences the smile onsets and offsets of the other partner. This time-to-event framing preserves information about the temporal dynamics of interaction. Research in this vein [13] indicates that infant and mother smile onsets and offsets tended to occur within 10 seconds of the other partner's actions between 1 and 6 months. However, mothers initiated smiles more predictably than infants, while infants terminated smiles more predictably than mothers. As in the current study, dyadic states were defined by presence and absence of infant and adult smiling (e.g. both partners smiling or only infant or only mother smiling). In any given dyadic smiling state, infant smile offsets were more predictable than infant smile onsets, suggesting that infants were more likely to end than to begin a smile. However, this pattern changed developmentally. Infant smile initiations became more predictable over the first 6 months of life, while their smile terminations became less predictable, suggesting an increasing infant propensity for smiling [13]. But little is known about the degree to which the infant propensity to terminate and initiate smiles changes developmentally with different social partners.

### (c) The present study

The goal of the current research was the detection of developmental changes in the rate at which infant and adult smiles began and ended in dyadic states defined by their smiling. Specifically, the presence and absence of infant and adult smiles created four dyadic states (i.e. both smiling, only infant smiling, only adult smiling and neither smiling). We modelled the rates of transitions between these states, which are owing to infant and adult smile onsets and offsets. These models directly tested whether infants, from 4 to 8 months, show increases or decreases in their smiling in the presence of a partner's smile, and whether these developmental patterns differ by partner.

To address limitations related to quantifying uncertainty in complex models with multiple levels of dependency, we employed multilevel survival (time-to-event) analysis of recurrent states via Poisson generalized additive mixed models within a multi-model inference framework to model the temporal coordination of infant and adult smiling. Smile onsets and offsets are discrete occurrences distributed across continuous time. Survival analyses, the core of the current approach, are used in analyses of infant behaviour [42] and dyadic interactions [43], and epidemiology [44,45]—where the focus is on modelling factors that accelerate or delay the occurrence of events. Treating smile onsets and offsets as discrete events with time-varying rates of occurrence (i.e. proportional hazards models) allowed us to ask whether the presence of adult smiling influenced the rate at which infants began and ended their smiles—and, reciprocally, whether the presence or absence of an infant smile influenced the rate at which adult partners began and ended smiles. By directly quantifying how each partner's current behaviour influenced the other's smile onsets and offsets across all possible timescales—avoiding fixed-lag specifications—we tested whether interaction is bidirectional. By modelling transition rates among joint states of infant and adult smiling while accounting for partner-specific patterns and developmental change, we provide insights into how infant coordination of positive affect develops early in life.

We video-recorded face-to-face interactions of 58 infants with their mothers, fathers and an unfamiliar adult stranger at 4 and 8 months, coding onset and offset times of infant and adult smiles using microanalytic methods validated in prior work with this sample [29]. The study addresses five research questions:

— *Baseline comparisons*. To shed light on the proximate causes of emotional expression production, we asked whether infants exhibit more rapid smile onsets and slower smile offsets in the presence of adult smiling. To shed light on the ultimate causes of early positive expressions (their effects), we examined the interpersonal consequences of infant smiling for infants' adult partners. That is, does the presence of an infant smile influence the rate at which adults start and end smiles? To understand infant smiling in an interactive context, we ask how infant rates of initiating, reciprocating and maintaining smiles compare with those of their adult partners.
— *Developmental effects*. As the proximate causes and social consequences of infant smiles may change developmentally, we asked whether the dependence of infant smile onsets (and offsets) on the presence of adult partner smiling changed from 4 to 8 months. Likewise, we asked whether the dependence of partner smiles on the presence of an infant smile changed with infant age. More generally, we asked whether infant rates of initiating, reciprocating and ending smiles change with age (and whether the same is true for infants' adult partners)?
— *Partner effects*. As the causes and consequences of infant smiles may vary by infant partner, we asked whether the dependence of infant smile onsets (and offsets) on the presence of partner smiling differed in interactions with mother, father and strangers. More generally, we asked whether infant rates of smile initiation, reciprocation and maintenance depended on the identity of the infant's adult partner (mother, father or stranger)?
— *Gender effects* (exploratory). Emotion regulation may differ by child gender [46,47]. Consequently, we explored gender effects by asking whether boys and girls initiate, reciprocate and maintain terminate smiles at different rates?
— *Statistical interaction effects*. Finally, we asked whether there were interactions between age, partner and infant gender in how infants and adults initiate, maintain and terminate smiles? For example, the possibility that mothers interact differently with boys versus girls [48] suggests the possibility of interaction effects between child gender and parent gender. More generally, age-by-partner effects would suggest that infant initiation, reciprocation and maintenance of smiles develop differently with parents than with a stranger.

## 2. Method

### (a) Participants

The sample consisted of 58 infants (25 girls) and their parents, recruited as part of a longitudinal study on social-communicative development over the first 2 years (My-BEST: Babies' Emotional & Social Trajectories). The present study focuses on data from the 4- and 8-month assessments [21,29,42,49]. Families were recruited in Amsterdam and surrounding areas through childcare centres, pregnancy courses, family-friendly locations (e.g. cafes with play areas, maternity shops) and advertisements in magazines and on social media. Parental eligibility criteria included having a 4-month-old infant who was the biological child of at least one of the parents. Infants were required to participate in at least one of the two scheduled home visits and to have at least one older sibling.

Infant age at the first home visit ranged from 111 to 143 days (*M* = 126.42, s.d. = 7.93) and at the second home visit from 227 to 272 days (*M* = 250.58, s.d. = 9.67). Mothers and fathers had mean ages of 34.5 and 37.9 years, respectively. Most parents were Dutch (91.4% of mothers; 84.2% of fathers). At the 4-month assessment, 55.2% of mothers were employed part-time and 19.0% full-time. Among fathers, 25.9% were employed part-time and 63.8% were employed full-time. For additional sociodemographic characteristics (e.g. parental education, income), see Salvadon *et al.* [29].

Each infant was scheduled to be observed in face-to-face interactions with three different adult partners (mother, father and stranger) at both time points, yielding a potential 348 dyadic video observations. One infant participated with both her mothers, and the two observations with the non-biological mother were excluded. An additional 59 observations were missing owing to home-visit cancellations (*n* = 36), technical errors (*n* = 3) and extreme infant fussiness (*n* = 20). The final dataset included 287 dyadic video observations capturing infant–adult interactions from all 58 infants: 147 observations at 4 months (51 infants) and 140 observations at 8 months (53 infants). For a detailed breakdown of available video observations by infant age and interaction partner, see table S1 in the supplementary material.

This study was conducted according to the Declaration of Helsinki and approved by the Ethics Review Board of the University of Amsterdam, Faculty of Social and Behavioural Sciences (protocol 2016-CDE-7403). Written informed consent was obtained from both parents prior to participation. Families were compensated with a €10 voucher at each home visit and received a USB flash drive containing all video recordings at the completion of the final assessment of the longitudinal study (at 18 months).

### (b) Procedure

**Figure 1.**
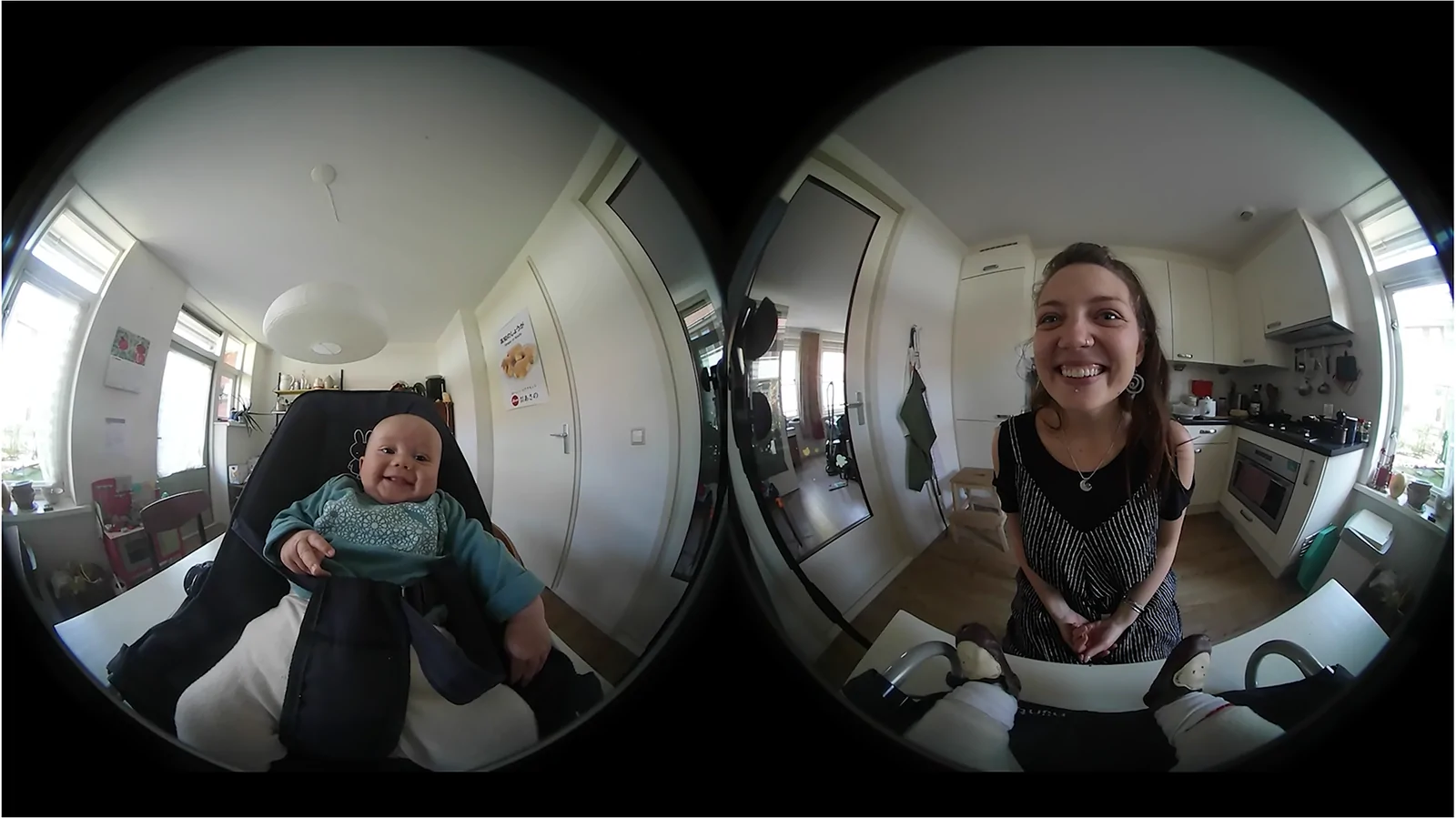
Video recording setup: interaction view. Example of the video recording from a face-to-face interaction between a 4-month-old infant and a stranger.

At 4 and 8 months, 2-minute infant face-to-face interactions were video-recorded separately for mothers, fathers and strangers (i.e. a female experimenter). Two-minute face-to-face interactions are common, especially when infants interact with multiple partners [50–52]. Each 2-minute face-to-face interaction with a partner—the focus of this article—was followed by an interaction with toys with the same partner. Interaction partner order was fully counterbalanced across families, with balanced distributions within each infant gender group. Infants were positioned in an age-appropriate infant seat facing the interaction partner. At 4 months, the infant was seated in an infant seat on the table, while at 8 months, the infant was seated in a high chair next to the table. A mobile high-definition dual-lens camera (Samsung GEAR 360°, 2016) captured both partners simultaneously in a high-resolution wide-angle split-screen video recording (3840 × 2160 pixels at 30 Hz; figure 1).

Parents were instructed to interact with their infant as they would typically do in everyday life and were informed they could interrupt the observation at any time if the infant experienced distress or tiredness. Experimenters (strangers) were instructed to remain visually attentive and emotionally positive, adjusting their engagement to match the infant's emotional state. There was no warm-up period with the stranger. Interactions occurred in a quiet room with no other person present. In the (rare) circumstance where separate rooms were not available owing to the home's layout, experimenters and the non-interacting parent remained in the same room but positioned out of the infant's sight.

### (c) Micro-analytic coding of behaviour

Infant and adult facial expressions were coded offline on a second-by-second basis using The Observer Video Analysis Software XT 14.0 (Noldus, Wageningen). Following established coding schemes [28,29], facial expressions were coded into three mutually exclusive categories for both infants and adults: positive, negative and neutral. Our focus was on positive facial expressions and specifically smiles. Smiles were operationalized as zygomatic major contraction (lip corner puller, Action Unit (AU12)) that either did or did not involve the Duchenne marker (eye constriction, AU6, cheek raiser) and mouth opening (AU25, AU26 or AU27). All smiles occurring during the face-to-face interactions were considered social smiles because infant smiles tend to overlap periods of gazing at the partner's face [11,53]. Coding captured onset and offset times of smiles for both infant and adult. Data were exported for each member of the dyad such that each second was characterized as either 0 (smile absent) or 1 (smile present).

### (d) Reliability of the manual coding

Coding was performed by six trained coders. Coders were required to reach a minimum inter-rater reliability of Cohen's kappa greater than or equal to 0.70 with the senior coder before independently coding videos. The senior coder then double-coded approximately 15% of the infant dataset that had been coded by the trained coders. Kappa coefficients were 0.83 at 4 months and 0.89 at 8 months.

### (e) Validation of the manual coding

The reliability of human coding of adult facial expressions was not assessed. Instead, building on prior work with the infant sample [49], we validated manual coding of adult and infant smiling behaviours against PyAFAR (Python-based Automated Facial Action Recognition [54–56]), an automated facial action unit detection system that extracts frame-by-frame occurrence probabilities and intensities of facial action units based on the Facial Action Coding System (FACS [57]), using the adult and infant modules, respectively.

Validation analyses were conducted on a subsample of 42 videos (14.5% of the 289 manually coded videos). We focused on AU12 (lip corner puller) and AU6 (cheek raiser, which constricts the eyes), which are the primary action units associated with Duchenne and non-Duchenne smiles. We computed mean occurrence probabilities for AU12 and AU6 for both infants and adults (see [49]). For adults only, PyAFAR also provided intensity estimations (ranging from 0 to 5, where 0 is non-existent and 5 is maximum) for AU12 and AU6, reflecting the magnitude of AU activation. To assess agreement between PyAFAR-detected action units and manually coded smiles, we examined how well PyAFAR AU12 and AU6 probabilities (and, for adults, AU12 and AU6 intensities) predicted manually coded facial expression (smile versus no-smile) using two metrics, area under the curve (AUC) and F1 scores. AUC reflects the model's ability to discriminate between smile and non-smile episodes, while F1 represents the balance between precision (the proportion of PyAFAR smiles that were manually coded smiles) and recall (the proportion of manually coded smiles detected by PyAFAR). Agreement was good for both infants (AUC = 0.79, F1 = 0.74) and adults (AUC = 0.80, F1 = 0.74), indicating that manual coding captured smiling behaviours as validated against an independent automated detection system.

### (f) Analytic strategy

To assess how infants and their adult partners coordinated smiling, and how this coordination was affected by infant age, interaction partner and infant gender, we considered joint combinations of infant and adult smile states as forming a recurrent transition network. We modelled rates of transition among joint combinations of infant and adult smile states (e.g. infant smiling while adult smiling → infant smiling while adult not smiling) using multilevel time-to-event (survival) analysis via Poisson generalized additive mixed models [58].

We defined four possible dyadic states based on the absence (0) or presence (1) of smiling: neither partner smiling (00), only infant smiling (01), only adult smiling (10) and both partners smiling (11). For each observation, we constructed a 4 × 4 matrix of transition rates among these states (see supplementary material, table S2). The 16 cells of this transition-rate matrix have 8 degrees of freedom, with each degree of freedom representing the rate of a specific transition within a brief time period, denoted by a term with a single lambda (*λ*). Rates in the other eight cells (near-simultaneous double transitions and no-transitions yet) are calculated from these eight single-transition rates. See supplementary material for details.

### (g) Modelling how transition rates depend on other predictors

All statistical analyses were conducted in R [59]. Fitting the full suite of statistical models simultaneously was conducted using package *rslurm* [60] on a high-performance computing cluster in R v. 4.4.1, and post-processing was conducted on an Apple Macintosh M1 running R v. 4.4.2. Data and code are available via Figshare at https://doi.org/10.6084/m9.figshare.30743000 and GitHub at https://github.com/acobolew/infant-adult-smile.

We modelled how the eight independent (single) transition rates depended on time since last transition, infant age, interaction partner and infant gender using the *bam* function in the R package *mgcv* [61,62]. We used a proportional hazards model where each transition rate was modelled as a baseline hazard with multiplicative effects of predictors on hazards (additive effects of predictors on log-hazards). The model included:

— The ‘baseline fixed effect’ of how each rate might differ by time since the last transition (baseline hazard rate). We modelled the log of each baseline hazard as a smooth function of time since the last transition.
— The ‘dyad fixed effects’ of how each rate might differ by interaction partner (mother, father, stranger), infant age (4 versus 8 months), infant gender, and the two-way and three-way interactions among these predictors.
— The ‘random intercept’ variation of each transition rate by infant (capturing individual differences in baseline transition rates).
— The ‘random slope for infant age’ variation which captures how each rate's change between 4 and 8 months might differ by infant (some infants may show larger developmental changes than others).
— The ‘random slope for interaction partner’ variation of how each rate's change across interaction partner types might differ by infant (some infants may show larger partner-specific differences than others).

### (h) Multi-model inference

We evaluated 19 models representing all well-formed combinations of the three dyad main effects and their interactions (see supplementary material, table S3, for full model set). Using an information-theoretic multi-model inference framework [63], we calculated model weights based on the Akaike information criterion corrected for small sample sizes (AICc) via the R package *MuMIn* [64]. Rather than selecting a single ‘best’ model, we used model averaging to estimate the relative support for each predictor across all candidate models. This approach accounts for model uncertainty and avoids arbitrary dichotomous decisions about effect inclusion based on *p*-value thresholds.

We report model-averaged parameter estimates and 95% confidence intervals (CIs) for all effects. Additionally, we calculated the sum of Akaike weights across all models including each effect as an index of evidence for that effect (supplementary material, table S3). Weights exceeding 0.5 indicate at least modest support for including an effect; weights exceeding 0.9 indicate strong support [63]. The complement of an Akaike weight supporting an effect (one minus the effect's weight) represents the support for the null hypothesis that the effect can be excluded.

## 3. Results

### (a) Descriptive overview

Table S4 in the supplementary material presents the mean and standard deviations of the percentage of time spent smiling for infants and adults, by infant age, interaction partner and infant gender. Adults smiled more than infants. Overall, infant smiles occupied 26%–45% of interactions, while adult smiling occupied 72%–95% of interactions (see supplementary material, table S4). Table S5 in the supplementary material presents the median and interquartile ranges of the duration of infant and adult smile and non-smile states, as well as transition rates out of those states. Infant median smile durations were 4–5 seconds, while adult smile durations were 8–28 seconds.

### (b) Magnitude of support for specific fixed effects

We employed Poisson generalized additive mixed models within a multi-model inference framework to conduct multilevel survival analysis of recurrent states, modelling transition rates among the four joint smiling states (neither smiling, adult only, infant only, both smiling). Akaike weights favoured a model with main effects of infant age and interaction partner, no effect of infant gender, and no statistical interactions (weight = 0.93; supplementary material, table S3). Multi-model inference (fixed effect weights in supplementary material, table S6) revealed overwhelming support for infant age (weight > 0.99) and interaction partner (weight > 0.99). Infant gender received very little support (weight = 0.06) and showed minimal effects on coordination patterns (see supplementary material, table S7 and figure S3). Evidence disfavoured *any* interaction (cumulative weight across all interactions = 0.01), suggesting that effects of age, partner and gender operated independently across all transitions. We therefore focus on main effects in interpreting temporal coordination patterns.

**Figure 2.**
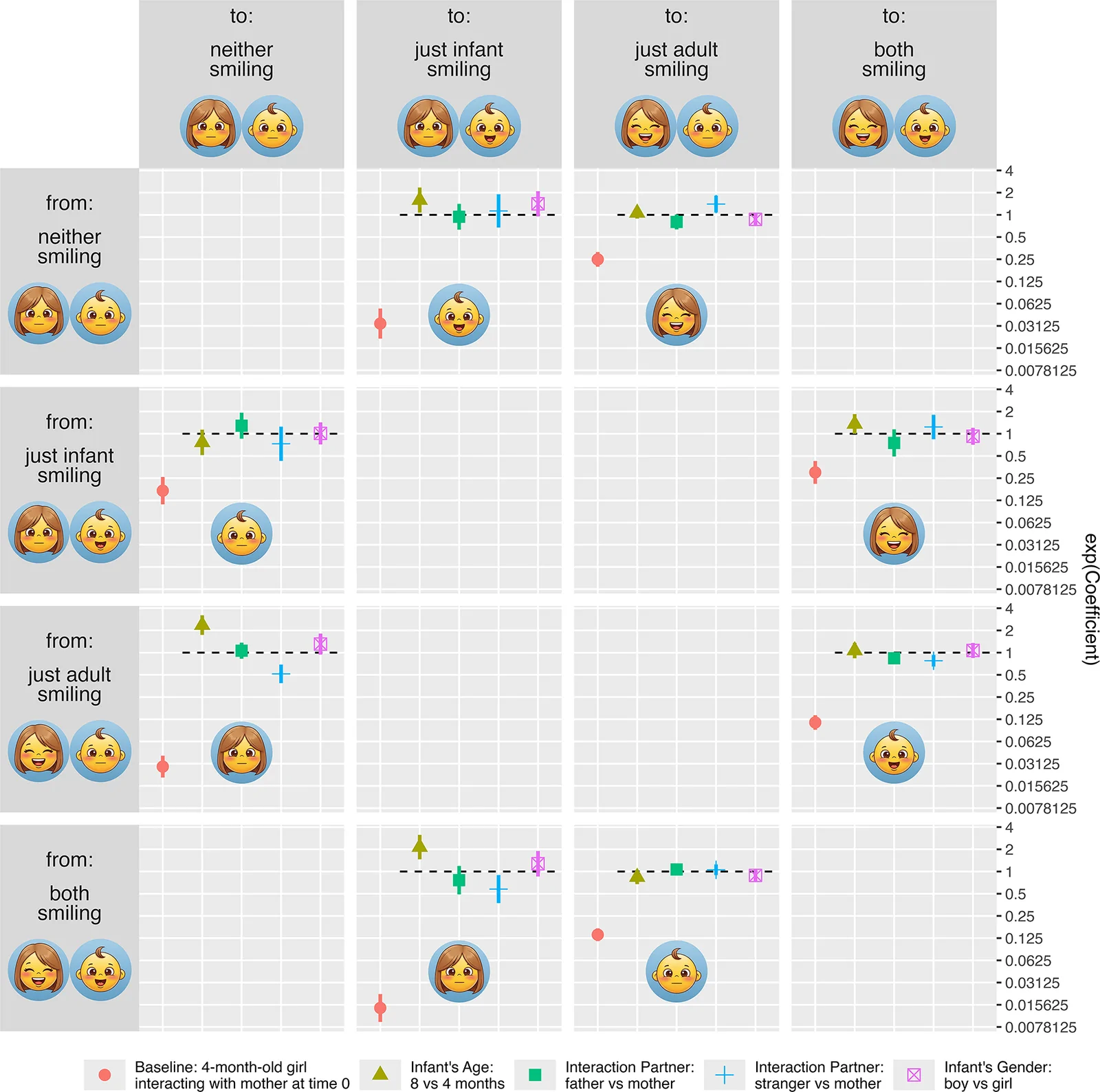
Model-averaged values of exp(coefficient) for baseline hazard rates and main effect HRs, for each of the eight single transitions. The prior states (‘from’) are described and illustrated in the left margin (rows), and the subsequent states (‘to’) are described and illustrated on the top margin (columns). The model-averaged baseline rates represent effects for 4-month-old girls interacting with their mothers. They are the left-most symbol in each panel. The main effect ratios for infant gender, age and adult partner are represented by the four right-most symbols in each panel. Dashed lines at HR of 1 indicate no difference from the baseline hazard rate. For example, the baseline hazard, in a dyad with a 4-month-old girl interacting with her mother, for the dyad to transition from neither partner person smiling (row 1) to only just the mother smiling (column 3) is 0.23 (the red circle in that panel). HRs in a panel are relative to the baseline hazard in the same panel. For the same transition (from neither partner smiling to only the adult smiling), the HR for stranger (versus mother) is 1.37 (cross symbol in the same panel, slightly above the dashed line that indicates a reference HR of 1). This indicates that when neither partner is smiling, strangers are slightly more likely than mothers to initiate a smile.

Figure 2 presents model-averaged baseline hazard rates at 0 time-in-state (i.e. immediately upon entering a state) for each of the eight single dyadic transitions. These baseline hazard rates represent effects for 4-month-old girls interacting with their mothers. They are the left-most symbol in each panel and range from 0.02 to 0.28. Contrasts among hazards are expressed as hazard ratios (HRs; supplementary material, table S7); contrasts among HRs are expressed as ratios of HRs (RHRs; supplementary material, table S9). The main effect HRs for infant gender, age and adult partner are represented by the four right-most symbols in each figure 2 panel. Each HR in each figure 2 cell represents how much the non-baseline hazard (e.g. 8-month-old, in the case of the filled triangles) differs from the baseline hazard, with elevated hazards in the non-baseline group indicated by HR greater than 1 and depressed hazards in the non-baseline group indicated by HR less than 1.

**Figure 3.**
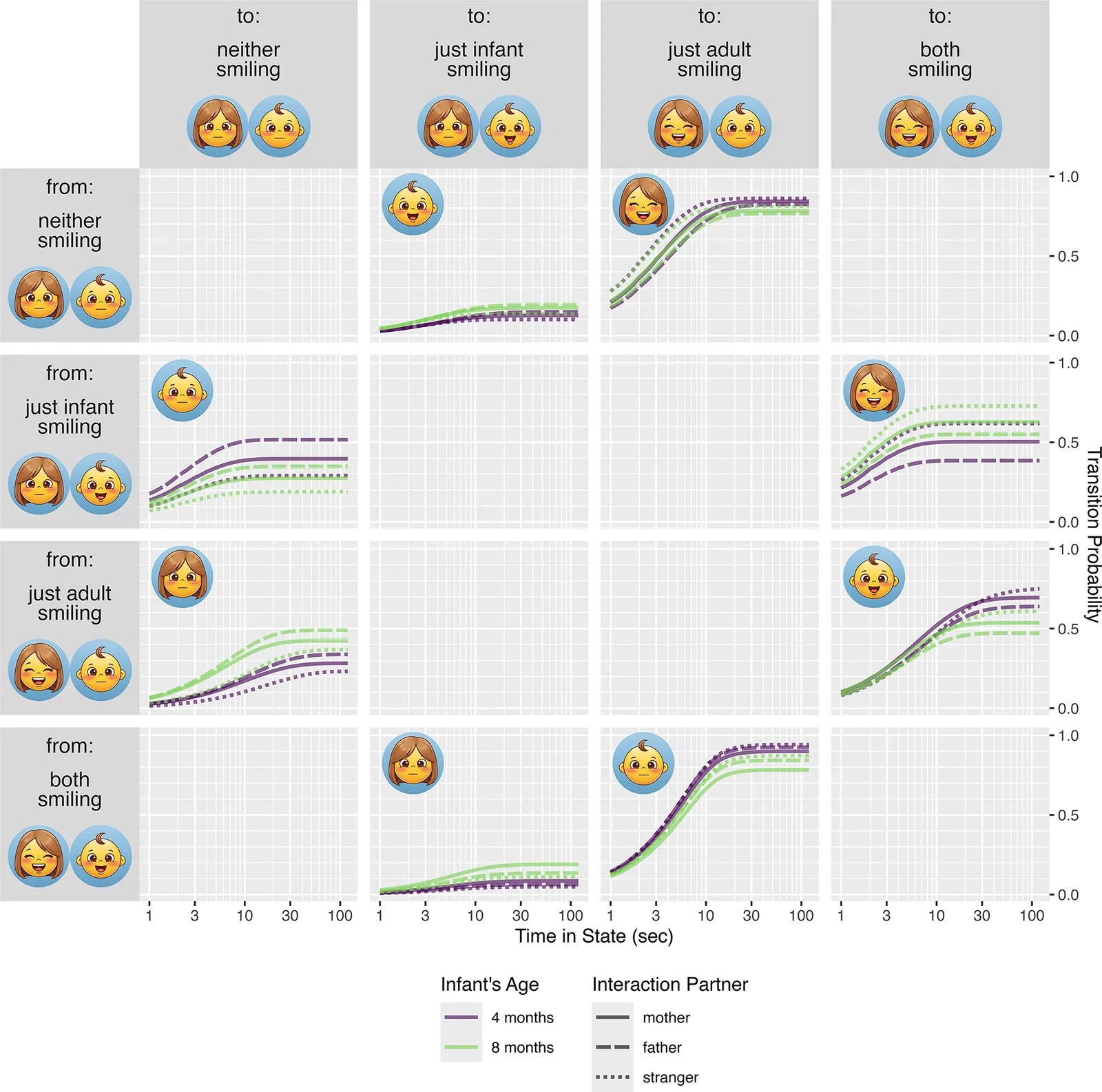
Cumulative incidence curves showing smiling patterns over time by infant age and interaction partner, for the eight single-transition cells (where a single partner initiated or ended a smile). This figure displays baseline curves for infant girls; curves for infant boys are almost identical (see supplementary material, figure S3). Cumulative incidence curves indicate the probability of a given dyadic transition as a function of time in the initial (‘from’) state. Median times for a curve are given by the number of seconds (*x*-axis) when a curve shows a 0.5 transition probability (*y*-axis). Separate curves are presented for infants at 4 and 8 months of age interacting with mothers, fathers and strangers (six curves per panel). Taking the transition from neither partner smiling (row 1) to only the adult smiling (column 3), for example, the baseline cumulative incidence curve is the solid purple line (4-month-olds interacting with mothers). The solid purple line starts at 0.20 after 1 second in state and increases to 0.81 by 120 seconds, indicating that, when neither partner is smiling, there is a 20% chance that the mother smiles immediately and an 81% chance that mothers ultimately smile before their 4-month-old infants. For the same transition (row 1, column 3), the cumulative incidence curve for 4-month-olds interacting instead with the stranger is represented by the dotted purple line. The difference between mother and stranger lines in the figure reflects the relative HR of strangers with respect to mothers (1.37) for the parallel transition (adult smiles) from figure 2.

While hazard rates in figure 2 represent the probability density of a transition at time 0, cumulative incidence curves represent the accumulating probability of the transition from one dyadic state to another over time, which occurs when a partner initiates, reciprocates or ends a smile. Figure 3 presents cumulative incidence curves for all values of time-in-state, by age and interaction partner. While figures 2 and 3 focus on the eight single transitions, figures S1 and S2 in the supplementary material present the full hazard curves and corresponding cumulative incidence curves and survival functions, respectively, for all values of time-in-state for all 16 cells—including the single-transition cells as well as the near-simultaneous double-transition cells and the no-transition cells.

### (c) Poisson multilevel survival analysis of smile transition rates

We describe and interpret the results for the eight single transition types (figures 2 and 3, rows 1–4). For each transition, we first present baseline hazard rates, establishing the fundamental temporal structure of infant–adult coordination. We then report developmental changes, followed by partner-specific patterns and infant gender effects. Model-averaged HRs and CIs for fixed-effect transitions and predictors are presented in supplementary material, table S7. Table S9 in the supplementary material presents contrasts between hazard rate coefficients as HRs and RHRs. We interpret HRs and RHRs whose 95% CIs do not overlap 1. The variance of random effects for the eight transition types and intraclass correlations (ICCs) are presented in supplementary material, table S8.

#### (i) Fixed effects

*Baseline coordination patterns. How do infant rates of initiating, reciprocating and maintaining smiles change in the presence of adult smiles and how do they compare with those of their adult partners?*

Coordination patterns are instantiated as baseline hazard rates (the filled red circles in figure 2) and illustrated by the differences between columns (panels) on a given row in figure 3 (see rows 1–8 of supplementary material, table S7). These hazards reflect the instantaneous chance of transitions between dyadic states at specific points in time, conditional on the events not having happened yet. The transitions between dyadic states reflect the smiling actions (onsets and offsets of infants and their adult partners). Contrasts among these data points—HRs are contained in the ‘baseline’ section of supplementary material, table S9, rows 1–28.

*Do infants exhibit more rapid smile onsets and slower smile offsets in the presence of adult smiling?* In the presence of adult smiling, infants began a smile (reciprocation) at almost three times the rate that they began a smile in the absence of adult smiling (initiation) (HR = 2.89, 95% CI (1.87, 4.47); figure 2, row 3, column 4 versus row 1, column 2). However, there was no difference in the rate at which infants ended smiles in the presence and absence of an adult smile (HR = 1.33, 95% CI (0.91, 1.94); figure 2, row 4, column 3 versus row 2, column 1). The results suggest that adult smiling primed quicker infant smile onsets but did not extend the duration of infant smiles.

*Do adults exhibit more rapid smile onsets and slower smile offsets in the presence of infant smiling?* The influence of infant smiling on adult behaviour followed a complementary pattern. The adult smile rate did not vary in the presence versus the absence of infant smiling (HR = 1.22, 95% CI (0.85, 1.74); figure 2, row 2, column 4 versus row 1, column 3), indicating no influence of infant smile on adult smile reciprocation and initiation rates. However, in the absence of an infant smile, adults ended their smiles at twice the rate they ended their smiles in the presence of an infant smile (HR = 2.02, 95% CI (1.28, 3.21); figure 2, column 2 versus row 3, column 1). Infant smiling did not prime adults to smile at a faster rate, but the infant smile served to extend the duration of adult smiles.

*How do infants compare with their adult partners with respect to initiating, reciprocating and maintaining smiles?* When neither partner was smiling (figure 2, top row), adults initiated smiles at almost six times the infant rate (HR = 5.75, 95% CI (3.64, 9.08)). When only one partner was smiling, adults reciprocated infant smiles at more than twice the rate infants reciprocated adult smiles (HR = 2.42, 95% CI (1.68, 3.47); figure 2, row 2, column 4 versus row 3, column 4). Likewise, when only one partner was smiling, infants ended their smiles at more than five times the adult rate (HR = 5.44, 95% CI (3.50, 8.46); figure 2, row 2, column 1 versus row 3, column 1). This pattern suggests that infant smiles draw adults into states of mutual positive affect more readily than adult smiles draw infants into these states. Strikingly, when both partners were smiling, infants stopped smiling at more than eight times the rate that adults stopped smiling (figure 2, row 4; HR = 8.29, 95% CI (5.45, 12.64)). Overall, these baseline findings reveal a pronounced asymmetry in smiling dynamics. Adults were more likely to initiate smiles first, reciprocate their partner's smiles more quickly and sustain smiles longer than infants, which created extended opportunities for infants to join adults in mutual smiling.

*Developmental changes. How do infant (and adult partner) patterns of smile initiation, reciprocation and maintenance change from 4 to 8 months?*

Developmental changes are indicated by the filled triangles in figure 2 and the purple- and green-coloured lines in figure 3 (see supplementary material, table S7, rows 9–16, for HRs, and table S9, rows 29–56, for the RHRs).

*Does the dependence of infant smiling rates on the presence of an adult smile (and vice versa) change developmentally?* Here, we ask whether differences between rates of infant smiling actions—for example, initiating versus reciprocating smiles—change with age. These are RHRs (see supplementary material, table S9). The infant proclivity to reciprocate adult smiles at a higher rate than infants initiated smiles did not change from 4 to 8 months (RHR = 0.69, 95% CI (0.21, 2.23)). Likewise, the ratio of the rates at which infants ceased solo and mutual smiles did not change with age (RHR = 1.17, 95% CI (0.66, 2.08)). With respect to the adult partner, the ratio of adult smile reciprocation and initiation did not change with age (RHR = 1.29, 95% CI (0.72, 2.31)). Likewise, the ratio at which adults ended their smiles in the presence and absence of an infant smile did not change with age (RHR = 0.90, 95% CI (0.10, 7.91)). In sum, the influence of adult smile presence on the rate of infant smile onsets and offsets (and the corollary influence of infant smiling on the adult) did not change with infant age.

*More generally, do infant smile onset and offset rates change with age?* When neither partner was smiling, 8-month-olds initiated smiling at more than one-and-a-half-times the rate of 4-month-olds (HR = 1.54, 95% CI (1.08, 2.19); figure 2, row 1, column 2). However, when only infants were smiling, 4- and 8-month-olds terminated their smiles at similar rates (HR = 0.73, 95% CI (0.53, 1.003); figure 2, row 2, column 1). Likewise, when only the adult partner was smiling, infants at 4 and 8 months reciprocated smiling at similar rates (HR = 1.06, 95% CI (0.85, 1.31); figure 2, row 3, column 2). Similarly, when both partners were smiling, infants at 8 months did not end their smiles at a lower rate than at 4 months (HR = 0.86, 95% CI (0.71, 1.03); figure 2, row 4), meaning that older infants did not significantly extend shared smiling bouts. In sum, 8-month-olds were more likely to initiate smiles in the absence of an adult smile, but they were not more likely to reciprocate adult smiles, or extend the duration of their own smiles, than 4-month-olds.

*Do adult partner rates of smile onsets and offsets change with infant age?* When only infants were smiling, the rate of adult reciprocation of infant smiles was slightly higher at 8 than 4 months (HR = 1.39, 95% CI (1.06, 1.81); figure 2, row 2). In addition, when smiling alone, adults ended their smiles with 8-month-olds at more than twice the rate at which they ended their smiles with 4-month-olds (HR = 2.35, 95% CI (1.76, 3.13); figure 2, row 3). The median duration of adult smiling alone was approximately 3 seconds when interacting with 8-month-olds and 5 seconds when interacting with 4-month-olds (supplementary material, figure S2, 3rd row), suggesting that adults left more time for younger infants than older infants to join in mutual smiling. Similarly, when both partners were smiling, adults ended their smiles with 8-month-olds at about twice the rate they ended their smiles with 4-month-olds (HR = 2.11, 95% CI (1.50, 2.96); figure 2, row 4). However, when neither partner was smiling, adult smile onset rates at 4 and 8 months did not differ (HR = 1.07, 95% CI (0.92, 1.25); figure 2, top row). In sum, adults modulated their smile duration and responsiveness as a function of infant age. Adults were more likely to terminate their smiles (both when smiling alone and when jointly smiling with the infant) with 8- than 4-month-olds, and were also more likely to reciprocate the smiles of older infants.

*Interaction partners. Do infant rates of smile initiation, reciprocation and maintenance depend on the identity of the infant's adult partner (mother, father or stranger)?*

Differences by interaction partner are indicated by the filled square (father versus mother) and the thin blue symbol (stranger versus mother) in figure 2, and the solid (mother), dashed (father) and dotted (stranger) lines in figure 3 (see supplementary material, table S7, rows 17–32). Differences in hazard rates (RHRs) by partner are described in supplementary material, table S9 (rows 57–84 for father versus mother, and rows 85–112 for stranger versus mother comparisons).

*Does the dependence of infant smiling rates on the presence of an adult smile (and vice versa) differ by partner?* The infant proclivity to reciprocate adult smiles at a higher rate than they initiated smiles did not differ by partner (with father versus mother RHR = 0.89, 95% CI (0.34, 2.32); with stranger versus mother RHR = 0.72, 95% CI (0.19, 2.71)). Likewise, the ratio of the rates at which infants ceased mutual smiles and solo smiles did not differ by partner (with father versus mother RHR = 0.87, 95% CI (0.57, 1.33); with stranger versus mother RHR = 1.50, 95% CI (0.52, 4.32)). With respect to adult smiling, the ratio of adults reciprocating versus initiating smiles did not vary by adult partner (father versus mother RHR = 0.99, 95% CI (0.41, 2.36); stranger versus mother RHR = 0.87, 95% CI (0.55, 1.39)). Likewise, the ratio at which adults ended their smiles in the presence and absence of an infant smile did not vary by partner (father versus mother RHR = 0.69, 95% CI (0.22, 2.16); stranger versus mother RHR = 1.15, 95% CI (0.62, 2.16)). In sum, there was no evidence that the influence of adult smiles on the infant proclivity to start and end smiles—or the same effect of infant smiling on adult smiles—differed depending on whether the infant was interacting with the mother, father or stranger.

*Do infant rates of smile initiation, reciprocation and maintenance vary by adult partner?* When only the adult was smiling, infants reciprocated stranger smiles at a lower rate than they reciprocated mother smiles (HR = 0.77, 95% CI (0.65, 0.90); figure 3, row 3) and also reciprocated father smiles at a lower rate than they reciprocated mother smiles (HR = 0.84, 95% CI (0.81, 0.996)). Notably, reduced smiling to strangers was specific to smile reciprocation. When neither partner was smiling, infants showed similar smile onset rates with mothers, fathers and strangers (figure 2, top row), father versus mother (HR = 0.95 95% CI (0.66, 1.36)); stranger versus mother (HR = 1.06, 95% CI (0.69, 1.62); figure 2, top row). Similarly, infants showed similar rates of smile offsets across partners, an index of how long infants maintained smiles with those partners. This was the case both when only the infant was smiling (figure 2, row 2;, father versus mother (HR = 1.23, 95% CI (0.88, 1.73); stranger versus mother (HR = 0.72, 95% CI (0.45, 1.13)) and in mutual smiling episodes (figure 2, row 4;, father versus mother (HR = 1.08, 95% CI (0.94, 1.24); stranger versus mother (HR = 1.08, 95% CI (0.94, 1.24); figure 2, row 4). Infants were more inclined to reciprocate mother smiles than smiles to other partners—suggesting that mother smiles were especially effective in eliciting joint smiling with the infant. However, infants were not more likely to maintain their smiles at the mother than at other partners, nor were they more likely to initiate smiles with one versus another partner. Thus, infant partner selectivity was only evident in the context of reciprocating ongoing adult smiles, while there were no reliable differences in other dyadic states.

*Do mothers, fathers and strangers differ in their initiation, reciprocation and maintenance of smiles with infants?* Adult partners showed distinct patterns of smile initiation and maintenance. When neither partner was smiling, strangers initiated smiles at higher rates than mothers (HR = 1.37, 95% CI (1.10, 1.71); figure 2, top row), who, in turn, initiated smiles at higher rates than fathers (HR = 1.23, 95% CI (1.01, 1.51)). When only the adult was smiling, strangers showed a lower rate of smile offsets than mothers, sustaining their smiles longer than mothers (HR = 0.50, 95% CI (0.39, 0.64); figure 2, row 3), while the rate of smile offsets did not distinguish mothers and fathers (HR = 1.08, 95% CI (0.85, 1.37)). Likewise, when both partners were smiling, strangers had lower rates of smiles offsets, sustaining their smiles longer than mothers (HR = 0.58, 95% CI (0.39, 0.86); figure 2, row 4); mothers and fathers did not show differences in their rates of smile offsets (HR = 0.74, 95% CI (0.51, 1.10)). When infants were smiling alone, the rate of mother, father and stranger smile reciprocation did not reliably differ (father versus mother (HR = 0.80, 95% CI (0.59, 1.08)); stranger versus mother (HR = 1.20, 95% CI (0.89, 1.62); figure 2, row 2). In sum, strangers initiated smiles *more* readily and sustained them longer than mothers, although mother smiles were most effective at eliciting infant smiles. Fewer differences were evident between mother–infant and father–infant smiling dynamics, with fathers initiating smiles the least.

*Gender effects. Do boys and girls initiate, reciprocate and maintain smiles at different rates?* Gender differences (indicated by the filled-in box in figure 2) were not detected. Model comparison provided little evidence for an infant gender effect (weight = 0.06; supplementary material, table S6), CIs for the HRs for all eight transitions overlapped 1 (figure 2, infant gender coefficients; supplementary material, table S7, rows 33–40), and survival curves for boys versus girls were nearly identical (see supplementary material, figure S3).

*Interaction effects. Are there interactions between age, partner and infant gender in how infants and adults initiate, reciprocate and maintain smiles?* Are there statistical interactions between age, partner and infant gender in how infants and adults initiate, reciprocate and maintain smiles? Evidence disfavoured any interaction (cumulative weight across all interactions = 0.013), supporting the hypothesis that effects of age, partner and gender operated independently across all transitions. Weight supporting infant age × infant gender was less than 0.01, as was the weight supporting interaction partner × infant gender. Even the best-supported single interaction (infant age × interaction partner) received little support (weight = 0.011). Evidence for the lack of interaction in each of these cases is 1 minus the weight in question.

#### (ii) Random effects

Table S8 in the supplementary material presents the variance of random effects of the baseline hazard rates and the residual variance of the baseline hazard rates and HRs for each of the eight single transitions. Table S8 in the supplementary material also presents the ICCs for these HRs, with high ICCs indicating that individual infants tend to maintain their position relative to other infants and low ICCs indicating less inter-individual stability. For individual infants, transition characteristics were more stable across 4 and 8 months (median ICC = 0.28 for infant age) than they were as infants interacted with mother, father and stranger (median ICC = 0.18 for interaction partner), meaning that a given dyad's smiling dynamics were more similar across developmental time than were a given infant's smiling dynamics across different partners at a given age. For both age and interaction partner, the highest ICC characterized the transition from only infant smiling to neither partner smiling, indicating that the rate at which infants ended their smiles when they were smiling alone reflects relatively stable individual differences that persist developmentally and across partners.

## 4. Discussion

Multilevel survival analysis of smile transitions indicated that positive affect coordination was characterized by bidirectional responsiveness between infants and adults that led the dyad towards somewhat different states. Comparisons of partners indicated that infants were more likely to create dyadic states in which only the adult was smiling through rapid termination of their own smiling and low rates of smile reciprocation. By contrast, adults were more likely to create joint smiling states through rapid initiation and reciprocation of infant smiling. These patterns changed developmentally. From 4 to 8 months, infants became increasingly likely to initiate smile exchanges. Over this period, adults became more likely to terminate their own smiles and reciprocate infant smiles, indicating a developmental reorganization of the dyadic system. Dyadic smile dynamics were relatively stable in this period, suggesting that infant and adult temporal patterning at 4 months constrained temporal patterning at 8 months. Infants exhibited selective smile responsiveness to their interactive partners. Strangers showed a greater predilection to initiate smiles than mothers and fathers, yet infants were more likely to reciprocate mother smiles than stranger and father smiles.

From the perspective of emotion socialization, our findings suggest that adults scaffolded infant smiles through rapid responsiveness (both initiating their own smiles and reciprocating infant smiles quickly) and persistence (sustaining smiles longer). Thus, adults consistently provided opportunities for infants to join and sustain mutual smiling states that may index shared joy. These opportunities constitute a temporal architecture that supports an infant's ability to reciprocate smiles (smile turn-taking) and to engage in mutual positive affect expressions. Smile turn-taking and joint smiling are lifelong competencies whose development is evident in the first 8 months of life. They are central to emotion communication and may support infant physiological regulation of positive affect [1,3,12,41,65].

Sequential analyses have shed light on infant–mother smiling contingencies but cannot consider the full time scale of dyadic smile transitions [35–39]. In the current study, infant and adult smiling formed a temporally linked dyadic system in which each partner's smiling created conditions that constrained the other, forming a temporal arc [3,12,66]. When neither partner was smiling, adults typically initiated smiles, which tended to elicit infant smiles, creating joint smiling states. Infants tended to end joint smiling states, leading adults to end their own smiles, creating joint non-smiling states.

Within the context of dyad interaction, multilevel survival analysis suggested that adult smiling appeared to be a proximal cause of infant smile onsets. But adult smiling did not influence infants to maintain the resulting joint smiling states. With respect to consequences, infant smiles did not lead adults to smile more quickly. This finding generalizes over partners and through 8 months the conditions in which infants may experience their smiles as sufficient but not necessary to elicit adult smiling [35,36]. By contrast, the presence of infant smiling sustained adults to continue joint smiling.

### (a) Bidirectional asymmetry in smile coordination

Face-to-face interaction research has documented bidirectional associations between infant and caregiver behaviour [12,25,41]. Substantially less attention has been devoted, however, to distinguishing how infants and caregivers influence one another by beginning and ending their smiles. Here, we concretize research showing a tendency for caregivers to elicit smiling from infants (smile reciprocation), and for infants to elicit non-smiling from their caregivers. Transitions between dyadic states may be conceived as moving in and out of synchrony where synchrony refers to matched states (both joint smiling and joint non-smiling) and asynchrony refers to states in which only one partner is smiling [67–69]. Infants and their adult partners, then, appear to show different paths in moving the dyad in and out of these synchronous and asynchronous states.

Compared to adults, who favoured smiling, infants favoured non-smiling states. This bidirectional pattern provides specificity to our understanding of the interactive development of social smiling [3]. Adults initiated smiles at seven times the infant rate, and reciprocated smiles at twice the infant rate. Thus, infant smiles were a more potent elicitor of adult smiling than the reverse. Specifically, infant smiles reliably drew adults into patterns of coordinated positive affect [35,36,70]. By the same token, adult smiling was more likely to elicit infant smiling than adult non-smiling. Although parental smiling may be more intuitive than intentional [71], adults appeared to time their smile initiations to maximize mutual positive affect. Ruvolo *et al.* [70] employed control theory to infer the most probable goals of infant and mother smiling actions between 1 and 4 months. They argue that mothers act to maximize periods of mutual smiling. The current findings suggest this pattern may continue through 8 months and generalize to fathers and strangers. It is important to note, however, that while adult smiles were associated with a higher rate of infant smile onsets, persistent adult smiling did not serve to prevent infants from terminating their smiles.

While the focus of the current study is not individual differences, high rates of adult smile initiation (when infants were not smiling) might be interpreted as intrusive. Likewise, high rates of adult reciprocation of infant smiles—and even high rates of adult termination of their own smiles when infants were not smiling—might be interpreted as overly responsive. The midrange model of socioemotional functioning suggests that very high—as well as very low levels of—maternal responsivity is associated with less optimal attachment outcomes [72,73]. Whether these patterns are evident when constrained to smiling dynamics—and whether they extend to fathers as well as mothers—are questions for future research.

Ruvolo *et al.* [70] argue that infants act to maximize states in which the mother is smiling, but the infant is not. This interpretation may be supported by current findings, such as the infant tendency to end mutual smiling by terminating their own smiles—which they did at almost eight times the adult rate—creating extended adult-only smiling states. An alternate interpretation, however, is that infant mutual smiling is a highly arousing but positive experience for infants that they regulate by ending their own smiles, creating cyclic experiences of increasing and decreasing positive affect. Importantly, infant smile termination does not indicate a break in interaction. Although not a feature of the current study, infant smiling is temporally coordinated with other expressive behaviours such as gazing at the parent's face and vocalizing. At both 3 and 6 months, infants tend to insert vocalizations into the course of a smile [53]. At 3 and 4 months, infant smiles tend to occur within the course of a gaze at the parent; by 6 and presumably 8 months, infants tend to disengage visually from the parent before ending their smiles [11,74,75]. Thus, although infants did not change their rate of smile offsets between 4 and 8 months, infant smile termination at 8 months is likely to signal a more definitive pause in engagement than at 4 months.

### (b) Developmental changes in interactive smiling

Infants exhibited an increase in social initiative, with 8-month-olds initiating smiles faster than 4-month-olds when neither partner was smiling. While infant smile initiations were less probable overall than adult initiations, the increase in infant smile initiations quantifies the degree to which older infants take more proactive roles in establishing positive affective exchanges [10,13]. These smile initiations may be a precursor to the increase in anticipatory smiling seen between 8 and 10 months of age in which infants begin to use smiles to communicate referentially [21,76].

The increase in infant smile initiation was the only infant-specific pattern that changed developmentally. The infant propensity to reciprocate rather than initiate smiles did not change with age. Likewise, the impact of adult smile presence on the rate of infant smile onsets and offsets (and the corollary influence of infant smiling on the adult) did not change with infant age, suggesting continuity in interactive influence on infant smiling between 4 and 8 months. In previous research, weekly observations of a modest number of infant–mother dyads indicated that infant smile offsets became less predictable with age [13]. By contrast, we found a change in the rate of smile offsets not for infants but for their adult partners.

Adults reciprocated the smiles of 8-month-olds at higher rates than those of 4-month-olds. Adults also interrupted joint smiling and their solo smiling with 8-month-olds at more than twice the rate they did so with 4-month-olds. When adults smiled alone, they provided 4-month-olds with almost twice as much time to engage in mutual smiling than they did with 8-month-olds (5 versus 3 seconds). However, adults did not show age-related differences in how quickly they initiated smiles. Viewed dyadically, adults engaged in quicker reciprocation and more abbreviated timing of smiling with older infants who were, in turn, more likely to initiate smiling when neither partner was smiling [1,5]. These findings suggest that developmental reorganization occurs as 8-month-olds become more likely to initiate smiles, smiles which adults become more likely to reciprocate. Overall, changes in each partner's smiling dynamics—infants initiating more proactively, adults reciprocating more quickly but holding their smiles for briefer durations—contribute to more dynamic smile turn-taking at 8 months [13].

Modelling revealed no evidence for an interaction of age and partner (or gender) effects. Thus, the development of interactive smiling was relatively independent of the partner with whom the infant interacted. The results provide a critical counterpoint to findings that infant–mother dyads develop distinct smiling dynamics over time [13]. However, the lack of evidence from fixed effects for the development of partner-specific smiling dynamics is tempered by random effects analyses that quantified the stability of individual differences in coordination, an approach rarely encountered in the literature. ICCs indicated that a given infant–adult dyad's interactive smiling dynamics were relatively stable from 4 to 8 months compared to the stability of infant smiling dynamics with different partners. The finding that within-dyad temporal stability over development was greater than between-partner variation suggests that parents and strangers exerted differing interactive influences on a given infant's smiling dynamics, especially those involved in joining and leaving joint smiling states. Infant smile terminations when smiling alone showed the highest stability both across developmental time and partner, suggesting relatively stable individual differences related to infant affect regulation.

### (c) Partner-specific coordination in infant–adult smiling

Infants exhibited graded partner-specific selectivity in smile reciprocation: infants were most likely to reciprocate mother smiles, less likely to reciprocate father smiles and least likely to reciprocate stranger smiles. Critically, the difference in infant smiling to mothers and strangers emerged only in reciprocating. Infants showed no partner differences when initiating smiles or maintaining mutual smiles. Adults displayed somewhat complementary partner-specific patterns. Strangers initiated smiles more rapidly than mothers and sustained those smiles longer both when smiling alone and during mutual smiling states. This pattern may reflect instructions to strangers to be emotionally positive whenever appropriate. Together, these findings confirm that early smile coordination is partner-sensitive as early as 4 months [30,31]. Despite strangers' heightened efforts (initiating smiling fastest and sustaining smiles longest), infants were less likely to reciprocate stranger smiles than mother smiles. This dissociation suggests that adult smiling strategies and infant selective responding operate through distinct mechanisms shaped by familiarity and relationship history. Stranger elevated initiation and persistence probably reflect compensatory strategies when interaction history is limited [77], yet infant selectivity limited the effectiveness of stranger efforts. By contrast, mothers' more efficient elicitation of infant smiles—despite reduced smile initiation and persistence with respect to stranger smiles—suggests that their smiles carry contextually salient meaning rooted in accumulated interactive history and predictability [13,29,30,31].

When neither partner was smiling, mothers initiated smiles at a higher rate than fathers, which may reflect greater reported out-of-home work among fathers. Despite fathers' lower smile initiation rate, once a smiling episode was underway, fathers and mothers were indistinguishable in reciprocation rates, smile offset rates and the degree to which their smiling influenced infant behaviour. This suggests that mother and father interactions may offer rather functionally similar smiling environments for infants, even if the frequency with which fathers enter those episodes is lower. The finding is consistent with aggregate evidence showing higher overall smiling with mothers than fathers [25,26,28,29]. Prior work suggests that father–infant interaction is characterized by higher intensity, more arousing bursts of positive affect [23,25]. Capturing the *intensity* of adult and infant smiles in future work may help clarify mother–father differences in smile dynamics.

### (d) Innovations, limitations and future directions

This study advances understanding of the proximate causes, social consequences and interactive context of infant smiling through three innovations. First, including multiple partners per infant—something rarely achieved in infant research where designs typically focus on mother–infant interaction—allowed us to distinguish infant-general from relationship-specific patterns. Faster infant smile initiations at 8 months generalized across partners (an infant-general developmental change), while reciprocation propensity varied by partner (a relationship-specific pattern). This distinction has theoretical importance. It suggests that some aspects of infant positive expressivity (spontaneous smile production) are evident across partners and may reflect infant characteristics such as temperament [21,29,78], while others (responsive smile matching) are shaped by accumulated interaction history with specific partners [12,13,67].

A second innovation involved modelling transition rates among discrete joint states as continuous functions of time. This approach directly quantified how each partner's behaviour influenced the other's smile onsets and offsets, capturing the temporal texture—the rhythm and timing—of infant and adult initiations and responses to one another. Modelling real-time patterns of interaction through transition rates among discrete joint states offers broader utility as temporal coordination is fundamental to many developmental phenomena (e.g. joint attention, conversational turn-taking). By specifying ‘who does what when’ with high time resolution, survival analysis and related event-history methods can reveal mechanisms of mutual influence that coarser approaches obscure. As developmental science moves towards understanding processes and mechanisms, methods that preserve temporal structure while accounting for statistical dependencies are central.

A final innovation involved the use of information-theoretic multi-model inference that quantified uncertainty without arbitrary significance dichotomization by weighting models by their relative evidence (AICc weights). That is, effects that explained the data were supported not only by a single model but by a weighted combination of all possible models [63]. Rather than testing against a null hypothesis at a fixed alpha threshold, the weighted combination of models quantified the evidence both for and against the impact of age, partner and gender in shaping the development of infant interactive positive expressivity.

The present study has limitations that suggest avenues for future research. First, analyses focused on smile presence and absence but not the morphology and intensity of smiles (e.g. Duchenne versus non-Duchenne smiles), which may convey distinct social meanings and elicit different partner responses [18,73,79]. Future research could profitably ask how the morphology of infant and adult smiles is coordinated in time. Second, we focused exclusively on smiling without modelling other related communicative modalities such as gaze and vocalization [11,12,28,29]. Yet an infant smile accompanied by sustained gaze and vocalization may be produced in different circumstances and elicit different adult responses than a smile produced in isolation. Future research could ask whether multimodal signals show different transition dynamics than unimodal signals. Third, our relatively culturally homogeneous sample limits generalizing to populations with different parenting practices [80], face-to-face engagement norms [81], and display rules for positive affect [82]. Cross-cultural research is necessary to understand the variability of infant–adult smiling dynamics and their development. Finally, 4- and 8-month time points provided a developmental comparison, but could not capture finer trajectories and potential nonlinearities in how smile coordination evolved. Denser longitudinal designs (e.g. weekly or monthly observations) might better capture the development of infant positive expressivity over time. Likewise, little is known about how infant positive affect dynamics during brief face-to-face interactions (e.g. 2–3 minutes) compare to those that might be captured during longer interactions (e.g. 2–3 hours). Simultaneously estimating moment-to-moment coordination (seconds) and developmental reorganizations (months) over longer observations could reveal how infant positive emotion development dynamics develop over multiple timescales.

## 5. Conclusion

Modelling the real-time dynamics of infant positive affect expressivity during early social interactions with multiple adult partners specified findings of bidirectional asymmetric coordination common in the literature. Viewed as a dynamic dyadic system, adults were quick to initiate and reciprocate smiles while infants showed a proclivity to end solo and joint smiling states, down-regulating positive affect. On the other hand, infants did reciprocate adult smiling, a pathway to positive expressivity that is likely to provide infants with experiences of shared joy. Continuing this cycle, it was infants who typically terminated mutual smiling, which led their adult partners to also cease smiling. Between 4 and 8 months, infants became more likely to smile in the absence of an adult smile, taking on what had been the more exclusively adult role by initiating positive affective exchanges. Over the same developmental period, adults became more likely not only to reciprocate infant smiles but also to terminate their own smiling, supporting faster-paced smile dynamics. Although strangers initiated smiles at higher rates than mothers and fathers, infants were more likely to engage in mutual smiling with mothers—probably reflecting their shared history. Thus, the multi-partner design indicated that the infant propensity to reciprocate smiles may be relationship-specific, while the tendency to terminate solo smiles shows stability across partners and age. Overall, quantifying interactive temporal coordination between dyadic smiling states revealed mechanisms of mutual influence through which even young infants actively participate in creating the contexts for the development of their own positive affect.

## Supplementary Material



## Data Availability

Fully anonymized data and code for the analyses as published are available for public use and can be downloaded from doi:10.6084/m9.figshare.30743000 and any post-publication updates to code can be downloaded from https://github.com/acobolew/infant-adult-smile. The raw video data are stored in the data repository of the University of Amsterdam and are not shared in open access format to protect participants' confidentiality. Supplementary material is available online [83].

## Theme

One contribution of 19 to a theme issue ‘Mechanisms, development, phylogeny and functions of emotional expressions’.
