# Supplementary material for: Smiling in time: the interactive development of infant smiling with mothers, fathers and strangers
Source: Philos Trans R Soc Lond B Biol Sci. 2026 Sep 24;381(1959):20250142. doi: 10.1098/rstb.2025.0142 (PMC13601870; doi:10.1098/rstb.2025.0142)

Supplementary Materials A: Available Video Observations

Table S1. Number of Video Observations by Infant Age and Interaction Partner (*N* = 289)

**Age**	**Total Videos**	**Infant-Adult Dyads**	***Infant* with Mother Videos**	***Infant* with Father Videos**	***Infant* with Stranger Videos**	***M* (*SD*) video duration (s)**
4 months	148	51	49	50	49	121.24 (5.81)
8 months	141	53	48	46	47	113.35 (20.98)

Supplementary Materials B: Structure of Matrix of Transition Rates

We defined four possible dyadic states based on the absence (0) or presence (1) of smiling: neither partner smiling (00), only infant smiling (01), only adult smiling (10), and both partners smiling (11). For each observation, we constructed a 4 × 4 matrix of transition rates among these states (see Table S2). Each entry in this matrix is a hazard rate. The 16 cells of this transition-rate matrix have 8 degrees of freedom, with each degree of freedom representing the rate of a specific transition within a brief time period, denoted by a term with a single lambda (λ) in Table S2. Rates in the other 8 cells (i.e., no-transition yet and near-simultaneous double transitions) are calculated from these 8 single-transition rates. Transition rates for near-simultaneous double transitions (e.g., neither partner smiling to both partners smiling) were modeled as the product of each individual transition rate. Although not included in the results, double transitions contribute – at a level proportional to their low rates – to model fit and are included in Figure S1 and Figure S2 in Supplementary F as well as Figure S3 in Supplementary G (see negative diagonal cells).

The structure of the matrix of transition rates is as follows:

Off-diagonal cells (8 cells on neither the principal diagonal nor the negative diagonal): Represent single transitions, where either infant or adult smile changes state. For example, the rate of transition from neither smiling to only infant smiling (adult smile onset) is $\lambda_{00.01}$. These are the study results reported in the body of the manuscript.

Negative diagonal cells (4 cells, lower-left to upper-right): Represent double transitions, where both infant and adult smiles change simultaneously (that is, within the same second). For example, the rate of transition from neither smiling to both smiling is $\lambda_{00.11} = \lambda_{00.01}\lambda_{00.10} + O(\lambda_{00.01}^3 + \lambda_{00.10}^3)$, where the "big O" notation indicates a second-order approximation–that is, one that omits cubic or higher order terms, as they are negligible. (For clarity, the "big O" notation is omitted from Table S2.)

Principal diagonal cells (4 cells, upper-left to lower-right): Represent remaining in the current state (no transition). These are constrained such that each row sums to zero. For example, the rate of remaining in the state where neither partner is smiling (cell 00.00), shown in the top left cell, is $\lambda_{00.00} = -\lambda_{00.01} - \lambda_{00.10} - \lambda_{00.01}\lambda_{00.10} + O(\lambda_{00.01}^3 + \lambda_{00.10}^3)$.

The formulas in cells on both diagonals are accurate to second order.

The generalized additive mixed models are all proportional-odds models whereby each of the 8 single-transition hazards in this transition matrix vary smoothly over time (baseline hazard curve specific to that cell) and may also vary according to other fixed- and random-effects predictors. The hazard curves for the 8 off-diagonal cells can be computed from these 8 single-transition hazard curves as above.

Table S2. Matrix of Transition Rates

From State	To State			
	Adult Smile Off<br>Infant Smile Off<br>(00)	Adult Smile Off<br>Infant Smile On<br>(01)	Adult Smile On<br>Infant Smile Off<br>(10)	Adult Smile On<br>Infant Smile On<br>(11)
Adult Smile Off<br>Infant Smile Off<br>(00)	$-\lambda_{00.01}$<br>$-\lambda_{00.10}$<br>$-\lambda_{00.01}\lambda_{00.10}$	$\lambda_{00.01}$	$\lambda_{00.10}$	$\lambda_{00.01}\lambda_{00.10}$
Adult Smile Off<br>Infant Smile On<br>(01)	$\lambda_{01.00}$	$-\lambda_{01.00}$<br>$-\lambda_{01.00}\lambda_{01.11}$<br>$-\lambda_{01.11}$	$\lambda_{01.00}\lambda_{01.11}$	$\lambda_{01.11}$
Adult Smile On<br>Infant Smile Off<br>(10)	$\lambda_{10.00}$	$\lambda_{10.00}\lambda_{10.11}$	$-\lambda_{10.00}$<br>$-\lambda_{10.00}\lambda_{10.11}$<br>$-\lambda_{10.11}$	$\lambda_{10.11}$
Adult Smile On<br>Infant Smile On<br>(11)	$\lambda_{11.01}\lambda_{11.10}$	$\lambda_{11.01}$	$\lambda_{11.10}$	$-\lambda_{11.01}\lambda_{11.10}$<br>$-\lambda_{11.01}$<br>$-\lambda_{11.10}$

Supplementary Materials C: Model Set Details

Candidate Model Set

Among the three fixed effects (adult partner type, infant age, infant gender), we considered all well-formed models. We adhered to the marginality principle (hierarchical principle), whereby models containing interaction terms necessarily included all lower-order component effects. For instance, any model with a two-way interaction (e.g., Infant Age × Interaction Partner) also included both corresponding main effects. Similarly, the three-way interaction (Infant Age × Infant Gender × Interaction Partner) required inclusion of all three main effects and all three two-way interactions.

This yielded a candidate set of 19 models (see Table S3), including:

- Models with main effects only
- Models with main effects plus one or more two-way interactions (Interaction Partner × Infant Age, Interaction Partner × Infant Gender, Infant Age × Infant Gender)
- A model with the three-way interaction (Interaction Partner × Infant Age × Infant Gender) and all lower-order terms

Each main effect appeared in 14 of 19 models, each two-way interaction appeared in 6 of 19 models, and the three-way interaction appeared in 1 of 19 models (Table S3). This model set structure builds in a preference for parsimony, as simpler models (with fewer parameters) are more numerous in the candidate set.

Table S3. Obtained AICc weighting and presence/absence of fixed effects for each of 19 fitted models.

	Main Effects			Two-way Interactions			Three-way Interaction			
Fixed Effects in Model Formula	Age Group (A)	Interaction Partner (B)	Infant Gender (C)	A:B	A:C	B:C	A:B:C	AICc	ΔAICc	Akaike weight
A + B + C	✓	✓	✓					36754.88	0.00	0.7846
A + B + A:B	✓	✓		✓				36757.82	2.94	0.1806
A + B + C + A:C	✓	✓	✓		✓			36761.91	7.03	0.0233
A + B + C + A:B	✓	✓	✓	✓				36763.79	8.91	0.0091
B		✓						36767.23	12.35	0.0016
A + B + C + A:B + A:C	✓	✓	✓	✓	✓			36770.29	15.41	0.0004
A + B + C + B:C	✓	✓	✓			✓		36770.88	16.00	0.0003
B + C		✓	✓					36773.37	18.49	7.56E-05
A	✓							36773.74	18.86	6.29E-05
A + B + C + B:C + A:C	✓	✓	✓		✓	✓		36776.85	21.97	1.33E-05
A + C	✓		✓					36779.76	24.88	3.1E-06
A + B + C + A:B + B:C	✓	✓	✓	✓		✓		36782.18	27.30	9.25E-07
A + C + A:C	✓		✓		✓			36787.28	32.41	7.21E-08
B + C + B:C		✓	✓			✓		36787.37	32.49	6.92E-08
A + B + C + A:B + B:C + A:C	✓	✓	✓	✓	✓	✓		36788.03	33.15	4.97E-08
null model (no such fixed effects)								36792.56	37.68	5.15E-09
A + B + C + A:B + B:C + A:C + A:B:C	✓	✓	✓	✓	✓	✓	✓	36794.05	39.18	2.44E-09
C			✓					36798.37	43.49	2.82E-10
A + B	✓	✓						36816.12	61.24	3.94E-14

Note. Fixed effects in model formulas are designated as Infant Age (A), Interaction Partner (B), and Infant Gender (C). The model with only main effects had the lowest AICc. and is the focus of interpretation, though all coefficients presented in the manuscript average across all models.

Supplementary Materials D: Descriptive Statistics

Table S4. Mean and Standard Deviation of Infant and Adult Percentages of Time Spent Smiling, by Infant Age, Interaction Partner, and Infant Gender

**Age (months)**	**Infant Gender**	**Mother**		**Father**		**Stranger**	
		Infant	Adult	Infant	Adult	Infant	Adult
**4**	**Girls**	31.7 (23.2)	89.4 (13.5)	33.2 (24.1)	87.2 (18.5)	30.1 (24.3)	95.4 (4.7)
	**Boys**	39.6 (26.8)	83.4 (18.5)	25.4 (20.1)	72.2 (30.8)	30.6 (24.4)	93.8 (8.5)
**8**	**Girls**	41.0 (24.3)	81.7 (11.6)	27.5 (15.6)	77.3 (17.8)	26.0 (26.2)	87.4 (14.1)
	**Boys**	44.6 (24.2)	75.0 (15.3)	38.1 (21.3)	73.8 (22.2)	36.7 (25.4)	90.0 (9.6)

Note. Values are mean percentages with standard deviations in parentheses. The percentage of time smiling reported here is based on complete seconds of smiling (i.e., smile events lasting ≥1 second), which results in slightly higher values than those reported in our previous analysis of this dataset (Salvadori et al., 2022, 2024), where smile duration was calculated based on total duration including partial seconds. This difference reflects the methodological decision to analyze discrete smile events in full-second units for the survival analysis framework employed here.

Table S5. Median and Interquartile Range (IQR) of Individual Infant and Adult Smile State (Smile and No Smile) – and the corresponding Rate of Transition Out of that State per second – by Infant Age, Partner Who Begins/Ends Smile, and Interaction Partner

**Age (in months)**	**Partner who Begins/Ends Smiling**	**Smile State**	**Mother**		**Father**		**Stranger**	
			Duration in seconds	*Rate per second*	*Duration in seconds*	*Rate per second*	*Duration in seconds*	*Rate per second*
4	Infant	Smile	4 (4)	0.25 (0.19)	4 (5)	0.25 (0.36)	4 (6)	0.25 (0.38)
		No smile	6 (13)	0.17 (0.43)	8 (18)	0.13 (0.45)	7 (15)	0.14 (0.44)
	Adult	Smile	12 (33)	0.08 (0.17)	10 (34)	0.10 (0.17)	28 (69)	0.04 (0.08)
		No smile	3 (6)	0.33 (0.86)	3 (8)	0.33 (0.89)	2 (3)	0.50 (0.75)
8	Infant	Smile	4 (6)	0.25 (0.22)	5 (5)	0.20 (0.21)	5 (6)	0.20 (0.22)
		No smile	5 (11)	0.20 (0.42)	6 (13)	0.17 (0.43)	7 (15)	0.14 (0.28)
	Adult	Smile	8 (15)	0.13 (0.20)	8 (11)	0.13 (0.18)	15 (28)	0.07 (0.11)
		No smile	3 (4)	0.33 (0.80)	3 (5)	0.33 (0.83)	2 (3)	0.50 (0.75)

Note. Values are medians with interquartile ranges in parentheses. "Partner who Begins/Ends" indicates whether the infant or adult initiated the smile onset or offset. Duration reflects median time in the state before transitioning out. Rate reflects the median instantaneous transition rate per second. Note that the median rate of transition out of a given smiling or no smiling state per second is the inverse of the median duration of that state. E.g. A state with a median of 4 seconds has a median transition rate of 0.25 per second.

Supplementary Materials E: Multi-model Inference & Model Coefficients

Table S6. Obtained weights of each fixed effect.

**Fixed Effect**	**Akaike Weight Supporting Effect**[1]	**One Minus Weight**[2]
Infant Age	0.9983	0.0017
Interaction Partner	0.9999	0.0001
Infant Gender	0.8177	0.1823
Any 2- or 3-way interaction	0.2137	0.7863
Infant Age:Interaction Partner	0.1901	0.8099
Infant Age:Infant Gender	0.0237	0.9763
Interaction Partner:Infant Gender	0.0003	0.9997
Infant Age:Interaction Partner:Infant Gender	2.44E-09	1.0000

[1] Sum of Akaike weights of models that included effect
[2] Sum of Akaike weights of models that excluded effect

Table S7. Baseline hazard rates and hazard ratios for all between-session fixed effects

Row	Between-Session Predictor	Level of Predictor	Transition Code[3]	exp(Coefficient)[4]	95% CI Low	95% CI High	z
1	Baseline: 4-month-old girl with mother at time 0	Baseline	00.01	0.03	0.02	0.05	-30.16
2	Baseline: 4-month-old girl with mother at time 0	Baseline	00.10	0.25	0.20	0.31	-22.53
3	Baseline: 4-month-old girl with mother at time 0	Baseline	01.00	0.17	0.11	0.26	-16.16
4	Baseline: 4-month-old girl with mother at time 0	Baseline	01.11	0.30	0.21	0.43	-12.24
5	Baseline: 4-month-old girl with mother at time 0	Baseline	10.00	0.03	0.02	0.04	-32.44
6	Baseline: 4-month-old girl with mother at time 0	Baseline	10.11	0.11	0.09	0.14	-32.24
7	Baseline: 4-month-old girl with mother at time 0	Baseline	11.01	0.01	0.01	0.02	-35.66
8	Baseline: 4-month-old girl with mother at time 0	Baseline	11.10	0.14	0.11	0.17	-30.22
9	Infant Age	8-month-old	00.01	1.59	1.07	2.35	2.31
10	Infant Age	8-month-old	00.10	1.06	0.89	1.27	0.66
11	Infant Age	8-month-old	01.00	0.76	0.51	1.14	1.33

[3] We defined four possible dyadic states based on the absence (0) or presence (1) of smiling: neither partner smiling (00), only infant smiling (01), only adult smiling (10), and both partners smiling (11). Dyadic states before the dot separator indicate “from”, while states after the dot separator indicate “to”. For example, “00.01” indicates transition *from* neither partner smiling *to* only infant smiling (see Table S2).

[4] For baseline condition (rows 1-8), exp(Coefficient) is baseline hazard rate. For all other rows, exp(Coefficient) is the hazard ratio relative to the baseline for the corresponding Transition Code.

Row	Between-Session Predictor	Level of Predictor	Transition Code[3]	exp(Coefficient)[4]	95% CI Low	95% CI High	z
12	Infant Age	8-month-old	01.11	1.36	1.00	1.85	1.94
13	Infant Age	8-month-old	10.00	2.36	1.74	3.21	5.51
14	Infant Age	8-month-old	10.11	1.07	0.84	1.36	0.53
15	Infant Age	8-month-old	11.01	2.14	1.46	3.14	3.92
16	Infant Age	8-month-old	11.10	0.84	0.67	1.04	1.57
17	Interaction Partner	father	00.01	0.94	0.63	1.41	0.29
18	Interaction Partner	father	00.10	0.80	0.63	1.01	1.85
19	Interaction Partner	father	01.00	1.28	0.86	1.93	1.21
20	Interaction Partner	father	01.11	0.75	0.49	1.15	1.33
21	Interaction Partner	father	10.00	1.06	0.83	1.36	0.47
22	Interaction Partner	father	10.11	0.84	0.70	1.00	1.95
23	Interaction Partner	father	11.01	0.76	0.49	1.19	1.19
24	Interaction Partner	father	11.10	1.07	0.91	1.25	0.78
25	Interaction Partner	stranger	00.01	1.13	0.67	1.90	0.47
26	Interaction Partner	stranger	00.10	1.40	1.08	1.82	2.51
27	Interaction Partner	stranger	01.00	0.73	0.43	1.25	1.15

Row	Between-Session Predictor	Level of Predictor	Transition Code[3]	exp(Coefficient)[4]	95% CI Low	95% CI High	z
28	Interaction Partner	stranger	01.11	1.24	0.84	1.81	1.09
29	Interaction Partner	stranger	10.00	0.52	0.39	0.69	4.49
30	Interaction Partner	stranger	10.11	0.78	0.65	0.93	2.72
31	Interaction Partner	stranger	11.01	0.58	0.37	0.90	2.46
32	Interaction Partner	stranger	11.10	1.06	0.89	1.25	0.64
33	Infant Gender	boy	00.01	1.41	0.95	2.09	1.72
34	Infant Gender	boy	00.10	0.87	0.71	1.06	1.36
35	Infant Gender	boy	01.00	1.01	0.72	1.42	0.06
36	Infant Gender	boy	01.11	0.92	0.71	1.20	0.59
37	Infant Gender	boy	10.00	1.31	0.94	1.81	1.60
38	Infant Gender	boy	10.11	1.07	0.85	1.35	0.57
39	Infant Gender	boy	11.01	1.27	0.86	1.90	1.20
40	Infant Gender	boy	11.10	0.89	0.73	1.07	1.26
41	Infant Age : Interaction Partner	8-month-old : father	00.01	0.98	0.53	1.83	0.06
42	Infant Age : Interaction Partner	8-month-old : father	00.10	1.23	0.88	1.71	1.20
43	Infant Age : Interaction Partner	8-month-old : father	01.00	0.66	0.34	1.31	1.19

Row	Between-Session Predictor	Level of Predictor	Transition Code[3]	exp(Coefficient)[4]	95% CI Low	95% CI High	z
44	Infant Age : Interaction Partner	8-month-old : father	01.11	1.67	0.91	3.09	1.65
45	Infant Age : Interaction Partner	8-month-old : father	10.00	1.03	0.73	1.44	0.15
46	Infant Age : Interaction Partner	8-month-old : father	10.11	0.91	0.69	1.20	0.67
47	Infant Age : Interaction Partner	8-month-old : father	11.01	0.78	0.40	1.53	0.72
48	Infant Age : Interaction Partner	8-month-old : father	11.10	1.19	0.91	1.54	1.28
49	Infant Age : Interaction Partner	8-month-old : stranger	00.01	0.65	0.30	1.43	1.06
50	Infant Age : Interaction Partner	8-month-old : stranger	00.10	0.78	0.53	1.14	1.28
51	Infant Age : Interaction Partner	8-month-old : stranger	01.00	0.81	0.31	2.09	0.44
52	Infant Age : Interaction Partner	8-month-old : stranger	01.11	0.79	0.42	1.51	0.70
53	Infant Age : Interaction Partner	8-month-old : stranger	10.00	0.79	0.54	1.17	1.17
54	Infant Age : Interaction Partner	8-month-old : stranger	10.11	0.84	0.64	1.10	1.25
55	Infant Age : Interaction Partner	8-month-old : stranger	11.01	0.96	0.48	1.89	0.13
56	Infant Age : Interaction Partner	8-month-old : stranger	11.10	1.25	0.96	1.63	1.69
57	Infant Age : Infant Gender	8-month-old : boy	00.01	1.00	0.48	2.05	0.01
58	Infant Age : Infant Gender	8-month-old : boy	00.10	1.15	0.84	1.56	0.87
59	Infant Age : Infant Gender	8-month-old : boy	01.00	0.63	0.32	1.24	1.33

Row	Between-Session Predictor	Level of Predictor	Transition Code[3]	exp(Coefficient)[4]	95% CI Low	95% CI High	z
60	Infant Age : Infant Gender	8-month-old : boy	01.11	0.97	0.55	1.72	0.09
61	Infant Age : Infant Gender	8-month-old : boy	10.00	0.81	0.46	1.42	0.75
62	Infant Age : Infant Gender	8-month-old : boy	10.11	1.31	0.86	2.01	1.26
63	Infant Age : Infant Gender	8-month-old : boy	11.01	0.64	0.32	1.26	1.30
64	Infant Age : Infant Gender	8-month-old : boy	11.10	0.98	0.68	1.41	0.13
65	Interaction Partner : Infant Gender	father : boy	00.01	1.24	0.58	2.66	0.55
66	Interaction Partner : Infant Gender	father : boy	00.10	1.15	0.78	1.70	0.71
67	Interaction Partner : Infant Gender	father : boy	01.00	0.87	0.42	1.81	0.37
68	Interaction Partner : Infant Gender	father : boy	01.11	0.96	0.52	1.75	0.15
69	Interaction Partner : Infant Gender	father : boy	10.00	1.29	0.81	2.06	1.06
70	Interaction Partner : Infant Gender	father : boy	10.11	0.84	0.61	1.17	1.02
71	Interaction Partner : Infant Gender	father : boy	11.01	0.92	0.42	2.00	0.21
72	Interaction Partner : Infant Gender	father : boy	11.10	1.16	0.88	1.52	1.07
73	Interaction Partner : Infant Gender	stranger : boy	00.01	1.25	0.52	2.99	0.49
74	Interaction Partner : Infant Gender	stranger : boy	00.10	1.35	0.88	2.06	1.37
75	Interaction Partner : Infant Gender	stranger : boy	01.00	1.01	0.38	2.69	0.01

Row	Between-Session Predictor	Level of Predictor	Transition Code[3]	exp(Coefficient)[4]	95% CI Low	95% CI High	z
76	Interaction Partner : Infant Gender	stranger : boy	01.11	1.37	0.72	2.60	0.96
77	Interaction Partner : Infant Gender	stranger : boy	10.00	0.74	0.46	1.21	1.20
78	Interaction Partner : Infant Gender	stranger : boy	10.11	1.03	0.74	1.42	0.18
79	Interaction Partner : Infant Gender	stranger : boy	11.01	1.23	0.54	2.80	0.49
80	Interaction Partner : Infant Gender	stranger : boy	11.10	1.13	0.86	1.49	0.90
81	Infant Age : Interaction partner : Infant Gender	8-month-old : father : boy	00.01	2.56	0.59	11.02	1.26
82	Infant Age : Interaction partner : Infant Gender	8-month-old : father : boy	00.10	1.95	0.99	3.84	1.93
83	Infant Age : Interaction partner : Infant Gender	8-month-old : father : boy	01.00	0.40	0.08	1.93	1.15
84	Infant Age : Interaction partner : Infant Gender	8-month-old : father : boy	01.11	1.77	0.45	6.91	0.82
85	Infant Age : Interaction partner : Infant Gender	8-month-old : father : boy	10.00	1.13	0.57	2.28	0.35
86	Infant Age : Interaction partner : Infant Gender	8-month-old : father : boy	10.11	2.39	1.37	4.19	3.05
87	Infant Age : Interaction partner : Infant Gender	8-month-old : father : boy	11.01	2.06	0.51	8.28	1.02
88	Infant Age : Interaction partner : Infant Gender	8-month-old : father : boy	11.10	1.07	0.63	1.81	0.25
89	Infant Age : Interaction partner : Infant Gender	8-month-old : stranger : boy	00.01	6.47	1.17	35.75	2.14
90	Infant Age : Interaction partner : Infant Gender	8-month-old : stranger : boy	00.10	1.75	0.81	3.78	1.42
91	Infant Age : Interaction partner : Infant Gender	8-month-old : stranger : boy	01.00	1.03	0.11	9.76	0.03

Row	Between-Session Predictor	Level of Predictor	Transition Code[3]	exp(Coefficient)[4]	95% CI Low	95% CI High	z
92	Infant Age : Interaction partner : Infant Gender	8-month-old : stranger : boy	01.11	0.71	0.15	3.41	0.42
93	Infant Age : Interaction partner : Infant Gender	8-month-old : stranger : boy	10.00	1.77	0.81	3.86	1.44
94	Infant Age : Interaction partner : Infant Gender	8-month-old : stranger : boy	10.11	1.68	0.97	2.93	1.85
95	Infant Age : Interaction partner : Infant Gender	8-month-old : stranger : boy	11.01	1.49	0.33	6.72	0.52
96	Infant Age : Interaction partner : Infant Gender	8-month-old : stranger : boy	11.10	1.02	0.60	1.74	0.08

Table S8. Random-effect and residual variances and intraclass correlations (ICCs)

Between-Session Predictor	Transition Code[5]	σ^2_{child} (Random Effect Variance)	σ^2_{resid} (Residual Variance)	ICC[6]
Baseline	00.01	2.38E-06	NA	NA
Baseline	00.10	0.0066	NA	NA
Baseline	01.00	3.48E-08	NA	NA
Baseline	01.11	5.66E-08	NA	NA
Baseline	10.00	0.0007	NA	NA
Baseline	10.11	2E-07	NA	NA
Baseline	11.01	3.61E-07	NA	NA
Baseline	11.10	1.84E-07	NA	NA
Infant Age	00.01	0.0193	0.0516	0.2721
Infant Age	00.10	8.97E-05	0.0003	0.2315
Infant Age	01.00	1.94E-08	3.51E-08	0.3563
Infant Age	01.11	7.82E-08	1.78E-07	0.3046
Infant Age	10.00	0.0926	0.2358	0.2821
Infant Age	10.11	0.0573	0.1452	0.2830
Infant Age	11.01	0.0186	0.0562	0.2488
Infant Age	11.10	0.0359	0.0841	0.2993
Interaction Partner	00.01	0.0125	0.0542	0.1870
Interaction Partner	00.10	0.0041	0.0166	0.1988

[5] We defined four possible dyadic states based on the absence (0) or presence (1) of smiling: neither partner smiling (00), only infant smiling (01), only adult smiling (10), and both partners smiling (11).Dyadic states before the dot separator indicate "from", while states after the dot separator indicates "to". For example, "00.01" indicates transition *from* neither partner smiling *to* only infant smiling (see Table S2). For example, "00.01" indicates transition from neither partner smiling ("00" before the dot separator) to the infant smiling ("01" after the dot separator).

[6] For baseline hazard rate the ICC is not uniquely defined because variance of the random effect is completely confounded with the residual variance. In these cases the variance in the table is arbitrarily assigned to the random effect column.

Interaction Partner	01.00	8.05E-09	2.02E-08	0.2850
Interaction Partner	01.11	6.61E-07	2.56E-06	0.2049
Interaction Partner	10.00	0.0111	0.0865	0.1137
Interaction Partner	10.11	0.0021	0.0188	0.0985
Interaction Partner	11.01	0.0228	0.1046	0.1790
Interaction Partner	11.10	9.43E-05	0.0008	0.1019

Table S9. Table S9. Contrasts between fixed-effect coefficients: Hazard ratios (HR) and ratios of hazard ratios (RHR) for pairwise contrasts of transitions between dyadic states.

Row	Contrast Label[a]	Contrast Estimate	Contrast SE	z	p	exp of Contrast Est (HR or RHR)	CI_{lo} of HR or RHR	CI_{hi} of HR or RHR	reciprocal[d] exp of Contrast Est (HR or RHR)	CI_{lo} of reciprocal HR or RHR	CI_{hi} of reciprocal HR or RHR
	Baseline (4-month-old girl interacting w mother)[b]										
1	00.10-00.01	2.00	0.28	7.20	0.00	7.41	4.30	12.79	0.13	0.08	0.23
2	01.00-00.01	1.62	0.33	4.87	0.00	5.03	2.63	9.64	0.20	0.10	0.38
3	01.11-00.01	2.19	0.30	7.31	0.00	8.91	4.95	16.01	0.11	0.06	0.20
4	10.00-00.01	-0.17	0.29	-0.58	0.56	0.85	0.48	1.49	1.18	0.67	2.07
5	10.11-00.01	1.21	0.27	4.56	0.00	3.36	2.00	5.66	0.30	0.18	0.50
6	11.01-00.01	-0.87	0.32	-2.72	0.01	0.42	0.22	0.79	2.38	1.27	4.45
7	11.10-00.01	1.42	0.26	5.43	0.00	4.14	2.48	6.91	0.24	0.14	0.40
8	01.00-00.10	-0.39	0.24	-1.60	0.11	0.68	0.42	1.09	1.47	0.92	2.37
9	01.11-00.10	0.18	0.22	0.85	0.40	1.20	0.79	1.84	0.83	0.54	1.27
10	10.00-00.10	-2.17	0.22	-10.04	0.00	0.11	0.07	0.17	8.74	5.73	13.35
11	10.11-00.10	-0.79	0.16	-4.82	0.00	0.45	0.33	0.63	2.20	1.60	3.04
12	11.01-00.10	-2.87	0.25	-11.26	0.00	0.06	0.03	0.09	17.63	10.70	29.06
13	11.10-00.10	-0.58	0.15	-3.80	0.00	0.56	0.41	0.75	1.79	1.33	2.42

14	01.11-01.00	0.57	0.30	1.87	0.06	1.77	0.97	3.22	0.57	0.31	1.03
15	10.00-01.00	-1.78	0.28	-6.26	0.00	0.17	0.10	0.29	5.94	3.40	10.37
16	10.11-01.00	-0.40	0.25	-1.63	0.10	0.67	0.41	1.09	1.50	0.92	2.43
17	11.01-01.00	-2.48	0.31	-7.95	0.00	0.08	0.05	0.15	11.97	6.49	22.08
18	11.10-01.00	-0.20	0.24	-0.82	0.41	0.82	0.51	1.31	1.22	0.76	1.94
19	10.00-01.11	-2.35	0.25	-9.42	0.00	0.10	0.06	0.16	10.50	6.44	17.13
20	10.11-01.11	-0.97	0.22	-4.53	0.00	0.38	0.25	0.58	2.65	1.74	4.04
21	11.01-01.11	-3.05	0.28	-10.72	0.00	0.05	0.03	0.08	21.18	12.12	37.02
22	11.10-01.11	-0.77	0.21	-3.69	0.00	0.46	0.31	0.70	2.15	1.43	3.23
23	10.11-10.00	1.38	0.21	6.53	0.00	3.97	2.62	6.00	0.25	0.17	0.38
24	11.01-10.00	-0.70	0.27	-2.56	0.01	0.50	0.29	0.85	2.02	1.18	3.45
25	11.10-10.00	1.59	0.20	7.82	0.00	4.88	3.28	7.27	0.20	0.14	0.30
26	11.01-10.11	-2.08	0.25	-8.29	0.00	0.13	0.08	0.20	8.00	4.89	13.07
27	11.10-10.11	0.21	0.15	1.35	0.18	1.23	0.91	1.67	0.81	0.60	1.10
28	11.10-11.01	2.29	0.25	9.27	0.00	9.85	6.07	15.97	0.10	0.06	0.16
	8-month-old vs 4-month-old[c]										
29	00.10-00.01	-0.40	0.23	-1.78	0.07	0.67	0.43	1.04	1.49	0.96	2.32
30	01.00-00.01	-0.73	0.28	-2.64	0.01	0.48	0.28	0.83	2.07	1.21	3.57
31	01.11-00.01	-0.16	0.26	-0.60	0.55	0.86	0.51	1.42	1.17	0.70	1.95
32	10.00-00.01	0.40	0.25	1.58	0.11	1.49	0.91	2.44	0.67	0.41	1.10
33	10.11-00.01	-0.40	0.23	-1.72	0.09	0.67	0.43	1.06	1.49	0.95	2.33
34	11.01-00.01	0.30	0.27	1.09	0.28	1.35	0.79	2.31	0.74	0.43	1.27
35	11.10-00.01	-0.64	0.24	-2.68	0.01	0.53	0.33	0.84	1.89	1.19	3.01
36	01.00-00.10	-0.33	0.23	-1.45	0.15	0.72	0.46	1.12	1.39	0.89	2.16
37	01.11-00.10	0.25	0.18	1.36	0.17	1.28	0.90	1.82	0.78	0.55	1.11

38	10.00-00.10	0.80	0.18	4.32	0.00	2.22	1.55	3.19	0.45	0.31	0.65
39	10.11-00.10	0.01	0.15	0.04	0.97	1.01	0.75	1.35	0.99	0.74	1.34
40	11.01-00.10	0.70	0.22	3.20	0.00	2.01	1.31	3.10	0.50	0.32	0.76
41	11.10-00.10	-0.23	0.14	-1.65	0.10	0.79	0.60	1.05	1.26	0.96	1.67
42	01.11-01.00	0.57	0.29	2.01	0.04	1.77	1.01	3.10	0.56	0.32	0.99
43	10.00-01.00	1.13	0.25	4.49	0.00	3.08	1.89	5.04	0.32	0.20	0.53
44	10.11-01.00	0.33	0.23	1.45	0.15	1.40	0.89	2.19	0.72	0.46	1.12
45	11.01-01.00	1.03	0.27	3.85	0.00	2.80	1.66	4.72	0.36	0.21	0.60
46	11.10-01.00	0.09	0.25	0.38	0.70	1.10	0.68	1.78	0.91	0.56	1.47
47	10.00-01.11	0.55	0.23	2.46	0.01	1.74	1.12	2.70	0.58	0.37	0.89
48	10.11-01.11	-0.24	0.21	-1.15	0.25	0.79	0.52	1.18	1.27	0.85	1.91
49	11.01-01.11	0.46	0.26	1.78	0.07	1.58	0.96	2.60	0.63	0.38	1.05
50	11.10-01.11	-0.48	0.18	-2.63	0.01	0.62	0.43	0.88	1.62	1.13	2.31
51	10.11-10.00	-0.79	0.20	-3.92	0.00	0.45	0.30	0.67	2.21	1.49	3.28
52	11.01-10.00	-0.10	0.24	-0.40	0.69	0.91	0.56	1.46	1.10	0.68	1.78
53	11.10-10.00	-1.03	0.20	-5.16	0.00	0.36	0.24	0.53	2.81	1.90	4.16
54	11.01-10.11	0.69	0.23	3.01	0.00	2.00	1.27	3.15	0.50	0.32	0.79
56	11.10-10.11	-0.24	0.18	-1.36	0.17	0.79	0.56	1.11	1.27	0.90	1.80
57	11.10-11.01	-0.94	0.24	-3.97	0.00	0.39	0.25	0.62	2.55	1.60	4.04
	father vs mother[c]										
58	00.10-00.01	-0.16	0.24	-0.66	0.51	0.85	0.53	1.37	1.17	0.73	1.90
59	01.00-00.01	0.31	0.29	1.07	0.28	1.36	0.77	2.41	0.73	0.42	1.29
60	01.11-00.01	-0.23	0.30	-0.75	0.45	0.80	0.44	1.44	1.25	0.69	2.26
61	10.00-00.01	0.12	0.24	0.50	0.62	1.13	0.70	1.81	0.89	0.55	1.43
62	10.11-00.01	-0.12	0.22	-0.51	0.61	0.89	0.57	1.38	1.12	0.72	1.74

63	11.01-00.01	-0.21	0.30	-0.69	0.49	0.81	0.45	1.47	1.23	0.68	2.24
64	11.10-00.01	0.12	0.22	0.56	0.58	1.13	0.73	1.75	0.88	0.57	1.37
65	01.00-00.10	0.47	0.26	1.84	0.07	1.60	0.97	2.64	0.62	0.38	1.03
66	01.11-00.10	-0.07	0.22	-0.30	0.77	0.94	0.61	1.44	1.07	0.69	1.64
67	10.00-00.10	0.28	0.17	1.61	0.11	1.32	0.94	1.86	0.76	0.54	1.06
68	10.11-00.10	0.05	0.15	0.29	0.77	1.05	0.77	1.42	0.96	0.71	1.29
69	11.01-00.10	-0.05	0.27	-0.19	0.85	0.95	0.56	1.61	1.05	0.62	1.77
70	11.10-00.10	0.28	0.13	2.13	0.03	1.33	1.02	1.73	0.75	0.58	0.98
71	01.11-01.00	-0.54	0.35	-1.54	0.12	0.58	0.29	1.16	1.71	0.86	3.40
72	10.00-01.00	-0.19	0.24	-0.78	0.44	0.83	0.51	1.34	1.21	0.75	1.96
73	10.11-01.00	-0.43	0.22	-1.94	0.05	0.65	0.42	1.00	1.53	1.00	2.35
74	11.01-01.00	-0.52	0.29	-1.80	0.07	0.59	0.34	1.05	1.68	0.96	2.97
75	11.10-01.00	-0.19	0.23	-0.80	0.43	0.83	0.52	1.31	1.21	0.76	1.91
76	10.00-01.11	0.35	0.25	1.39	0.16	1.41	0.87	2.30	0.71	0.43	1.15
77	10.11-01.11	0.11	0.24	0.46	0.65	1.12	0.69	1.80	0.89	0.56	1.44
78	11.01-01.11	0.02	0.34	0.05	0.96	1.02	0.52	1.97	0.98	0.51	1.91
79	11.10-01.11	0.35	0.21	1.65	0.10	1.42	0.94	2.15	0.70	0.46	1.07
80	10.11-10.00	-0.24	0.16	-1.47	0.14	0.79	0.58	1.08	1.26	0.93	1.73
81	11.01-10.00	-0.33	0.26	-1.26	0.21	0.72	0.43	1.20	1.39	0.83	2.32
82	11.10-10.00	0.00	0.15	0.03	0.98	1.00	0.75	1.35	1.00	0.74	1.34
83	11.01-10.11	-0.10	0.24	-0.40	0.69	0.91	0.57	1.45	1.10	0.69	1.76
84	11.10-10.11	0.24	0.13	1.90	0.06	1.27	0.99	1.62	0.79	0.62	1.01
85	11.10-11.01	0.33	0.25	1.33	0.18	1.40	0.85	2.29	0.72	0.44	1.17
	stranger vs mother[c]										
86	00.10-00.01	0.21	0.28	0.76	0.45	1.24	0.71	2.15	0.81	0.46	1.40

87	01.00-00.01	-0.44	0.37	-1.19	0.23	0.65	0.32	1.32	1.55	0.76	3.17
88	01.11-00.01	0.09	0.30	0.29	0.77	1.09	0.60	1.98	0.92	0.50	1.66
89	10.00-00.01	-0.79	0.28	-2.78	0.01	0.46	0.26	0.79	2.19	1.26	3.82
90	10.11-00.01	-0.38	0.27	-1.40	0.16	0.69	0.41	1.16	1.46	0.86	2.46
91	11.01-00.01	-0.67	0.34	-1.96	0.05	0.51	0.26	1.00	1.96	1.00	3.83
92	11.10-00.01	-0.07	0.29	-0.23	0.81	0.93	0.53	1.66	1.07	0.60	1.90
93	01.00-00.10	-0.65	0.29	-2.21	0.03	0.52	0.29	0.93	1.92	1.08	3.41
94	01.11-00.10	-0.13	0.22	-0.58	0.56	0.88	0.58	1.35	1.13	0.74	1.74
95	10.00-00.10	-1.00	0.18	-5.49	0.00	0.37	0.26	0.53	2.72	1.90	3.88
96	10.11-00.10	-0.59	0.15	-3.89	0.00	0.55	0.41	0.75	1.80	1.34	2.43
97	11.01-00.10	-0.89	0.26	-3.44	0.00	0.41	0.25	0.68	2.42	1.46	4.02
98	11.10-00.10	-0.28	0.17	-1.62	0.10	0.75	0.54	1.06	1.33	0.94	1.87
99	01.11-01.00	0.53	0.35	1.51	0.13	1.69	0.86	3.34	0.59	0.30	1.17
100	10.00-01.00	-0.35	0.30	-1.16	0.25	0.71	0.39	1.27	1.42	0.79	2.56
101	10.11-01.00	0.06	0.28	0.21	0.83	1.06	0.61	1.85	0.94	0.54	1.64
102	11.01-01.00	-0.24	0.35	-0.67	0.50	0.79	0.40	1.57	1.27	0.64	2.51
103	11.10-01.00	0.37	0.29	1.26	0.21	1.44	0.81	2.56	0.69	0.39	1.23
104	10.00-01.11	-0.87	0.23	-3.84	0.00	0.42	0.27	0.65	2.40	1.53	3.75
105	10.11-01.11	-0.46	0.21	-2.26	0.02	0.63	0.42	0.94	1.59	1.06	2.38
106	11.01-01.11	-0.76	0.29	-2.59	0.01	0.47	0.26	0.83	2.14	1.20	3.80
107	11.10-01.11	-0.16	0.23	-0.70	0.48	0.85	0.55	1.33	1.17	0.75	1.82
108	10.11-10.00	0.41	0.17	2.46	0.01	1.51	1.09	2.09	0.66	0.48	0.92
109	11.01-10.00	0.11	0.27	0.43	0.67	1.12	0.67	1.88	0.89	0.53	1.50
110	11.10-10.00	0.72	0.18	3.91	0.00	2.05	1.43	2.93	0.49	0.34	0.70
111	11.01-10.11	-0.30	0.24	-1.23	0.22	0.74	0.46	1.19	1.34	0.84	2.15

112	11.10-10.11	0.31	0.14	2.27	0.02	1.36	1.04	1.77	0.74	0.56	0.96
113	11.10-11.01	0.60	0.24	2.47	0.01	1.83	1.13	2.95	0.55	0.34	0.88
	boy vs girl[c]										
114	00.10-00.01	-0.40	0.28	-1.42	0.16	0.67	0.39	1.16	1.49	0.86	2.57
115	01.00-00.01	-0.27	0.27	-1.00	0.32	0.76	0.45	1.30	1.31	0.77	2.25
116	01.11-00.01	-0.35	0.27	-1.27	0.20	0.71	0.41	1.21	1.41	0.83	2.41
117	10.00-00.01	-0.06	0.24	-0.27	0.79	0.94	0.59	1.50	1.07	0.67	1.70
118	10.11-00.01	-0.23	0.24	-0.96	0.34	0.80	0.50	1.27	1.25	0.79	1.99
119	11.01-00.01	-0.08	0.26	-0.32	0.75	0.92	0.55	1.53	1.09	0.65	1.81
120	11.10-00.01	-0.38	0.27	-1.41	0.16	0.68	0.40	1.16	1.46	0.86	2.49
121	01.00-00.10	0.12	0.20	0.63	0.53	1.13	0.77	1.66	0.88	0.60	1.30
122	01.11-00.10	0.05	0.16	0.32	0.75	1.05	0.78	1.43	0.95	0.70	1.29
123	10.00-00.10	0.33	0.24	1.40	0.16	1.40	0.87	2.23	0.72	0.45	1.14
124	10.11-00.10	0.17	0.16	1.05	0.29	1.19	0.86	1.63	0.84	0.61	1.16
125	11.01-00.10	0.31	0.26	1.23	0.22	1.37	0.83	2.26	0.73	0.44	1.21
126	11.10-00.10	0.02	0.13	0.12	0.90	1.02	0.79	1.31	0.98	0.77	1.27
127	01.11-01.00	-0.07	0.22	-0.34	0.74	0.93	0.61	1.42	1.08	0.70	1.65
128	10.00-01.00	0.21	0.24	0.88	0.38	1.23	0.77	1.97	0.81	0.51	1.29
129	10.11-01.00	0.05	0.20	0.24	0.81	1.05	0.71	1.54	0.95	0.65	1.40
130	11.01-01.00	0.19	0.25	0.75	0.45	1.21	0.74	1.98	0.83	0.50	1.36
131	11.10-01.00	-0.11	0.19	-0.58	0.56	0.90	0.62	1.30	1.11	0.77	1.61
132	10.00-01.11	0.28	0.24	1.20	0.23	1.33	0.84	2.11	0.75	0.48	1.20
133	10.11-01.11	0.12	0.17	0.70	0.49	1.13	0.80	1.58	0.89	0.63	1.24
134	11.01-01.11	0.26	0.25	1.04	0.30	1.30	0.79	2.13	0.77	0.47	1.26
135	11.10-01.11	-0.04	0.15	-0.23	0.82	0.97	0.72	1.30	1.04	0.77	1.39

136	10.11-10.00	-0.16	0.20	-0.80	0.42	0.85	0.57	1.27	1.18	0.79	1.75
137	11.01-10.00	-0.02	0.23	-0.08	0.93	0.98	0.62	1.55	1.02	0.64	1.62
138	11.10-10.00	-0.32	0.23	-1.38	0.17	0.73	0.46	1.14	1.37	0.88	2.16
139	11.01-10.11	0.14	0.23	0.63	0.53	1.15	0.74	1.80	0.87	0.56	1.35
140	11.10-10.11	-0.16	0.16	-0.99	0.32	0.86	0.63	1.17	1.17	0.86	1.59
141	11.10-11.01	-0.30	0.25	-1.20	0.23	0.74	0.46	1.21	1.35	0.83	2.19

[a]For example, contrast label 01.11-00.01 means the contrast of transition 01.11 (from only infant smiling (01) to both partners smiling (11)) minus transition 00.01 (from neither partner smiling (00) to only infant smiling (01)).

[b]Baseline coefficients are natural logarithm of hazards. The ratio of contrasts among baseline hazards (rows 1-28) are therefore hazard ratios (HR).

[c]Coefficients for 8-month-old vs 4-month-old, father-vs-mother, stranger-vs-mother, and boy-vs-girl are all natural logarithm of hazard ratios. The ratios among them (rows 29-141) are therefore ratios of hazard ratios (RHR).

[d]Reciprocal HR or reciprocal RHR is the ratio for the opposite contrast. For example, reciprocal HR for baseline contrast 00.10-00.01 is HR for baseline contrast 00.01-00.10. Likewise, the reciprocal RHR for 8-month-old-vs-4-month-old contrast for 00.10-00.01 is RHR for 8-month-old-vs-4-month-old contrast for 00.01-00.10.

Supplementary Materials F: Hazard and Cumulative Incidence Curves for all 16 cells

Figure S1. Absolute hazard curves showing smiling patterns over time by infant age and interaction partner. Boxed cells indicate the 8 single transitions (a single partner initiates or ends a smile). All cells off the principal diagonal (single transitions and double transitions) display hazard curves (conditional probability density that cell's transition is made at that instant, conditional on the transition not having been made yet, as a function of time since the state was entered). Cells on the principal diagonal ("no transition yet") display negative hazard rates (the sum of the other hazard curves in that row—negative hazard rates are plotted here because the actual hazard rates on the principal diagonal are negative so that each row can sum to 0, per Table S2 in Supplementary Materials B). Separate curves are presented for infants at 4 and 8 months of age interacting with mothers, fathers, and strangers (six curves per panel).

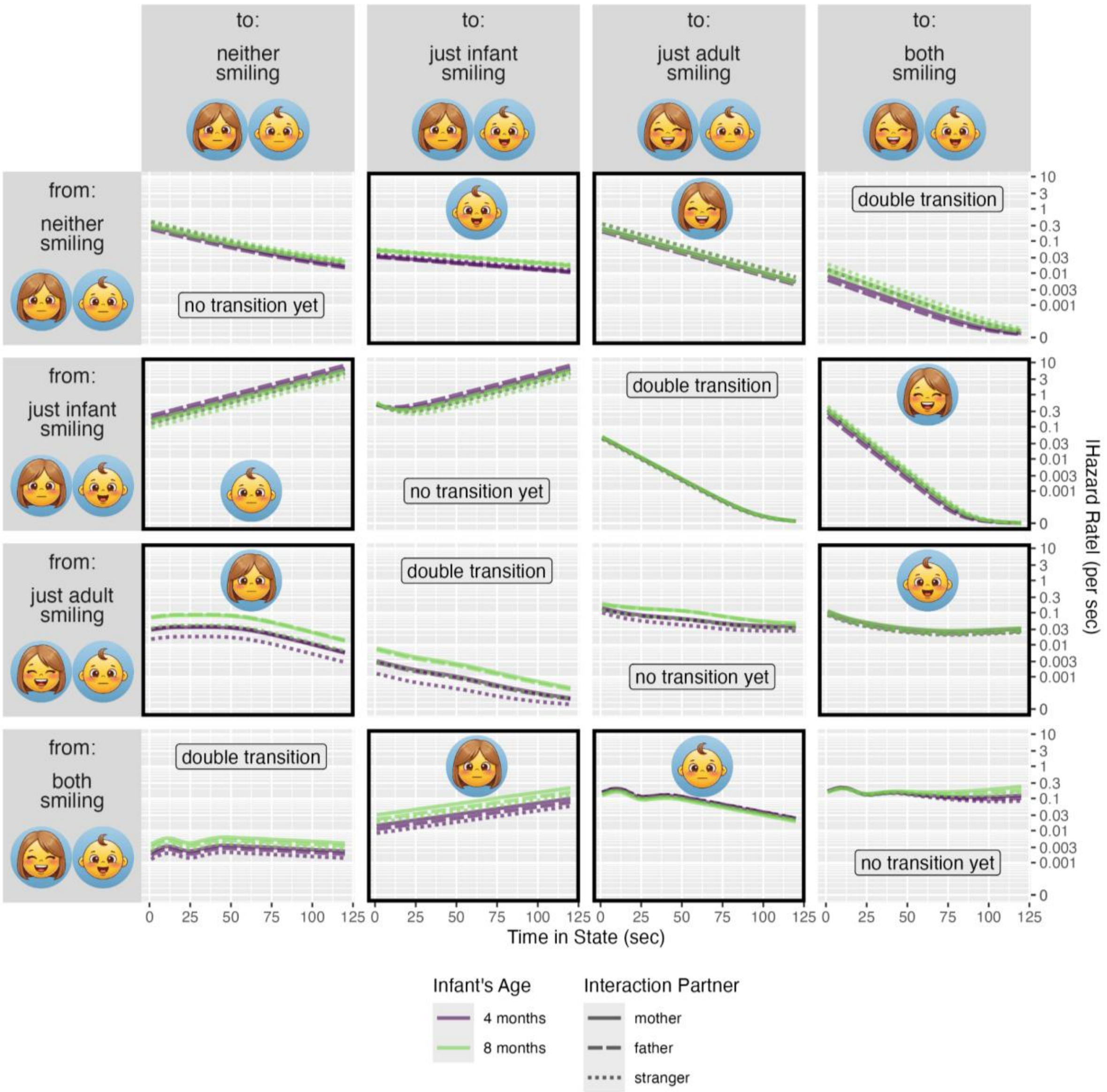

Figure S2. Survival and cumulative incidence curves showing smiling patterns over time by infant age and interaction partner. Boxed cells indicate the 8 single transitions (a single partner initiates or ends a smile). All cells on the principal diagonal ("no transition yet") display survival curves (probability that state has not yet changed, as a function of time since the transition into that state), and cells off the principal diagonal (single transitions and double transitions) display cumulative incidence curves (probability that that cell's transition is made, as function of time since transition into that state). Median times for a curve are given by the number of seconds (*x* axis) when a curve shows a 0.5 transition probability (*y* axis). Separate curves are presented for infants at 4 and 8 months of age interacting with mothers, fathers, and strangers (six curves per panel). Overall, the probability of no transition from a given state declines to 0 over time. The probability of single transitions (an infant or adult smile onset or offset) increases over time. The probability of double transitions is extremely low over time.

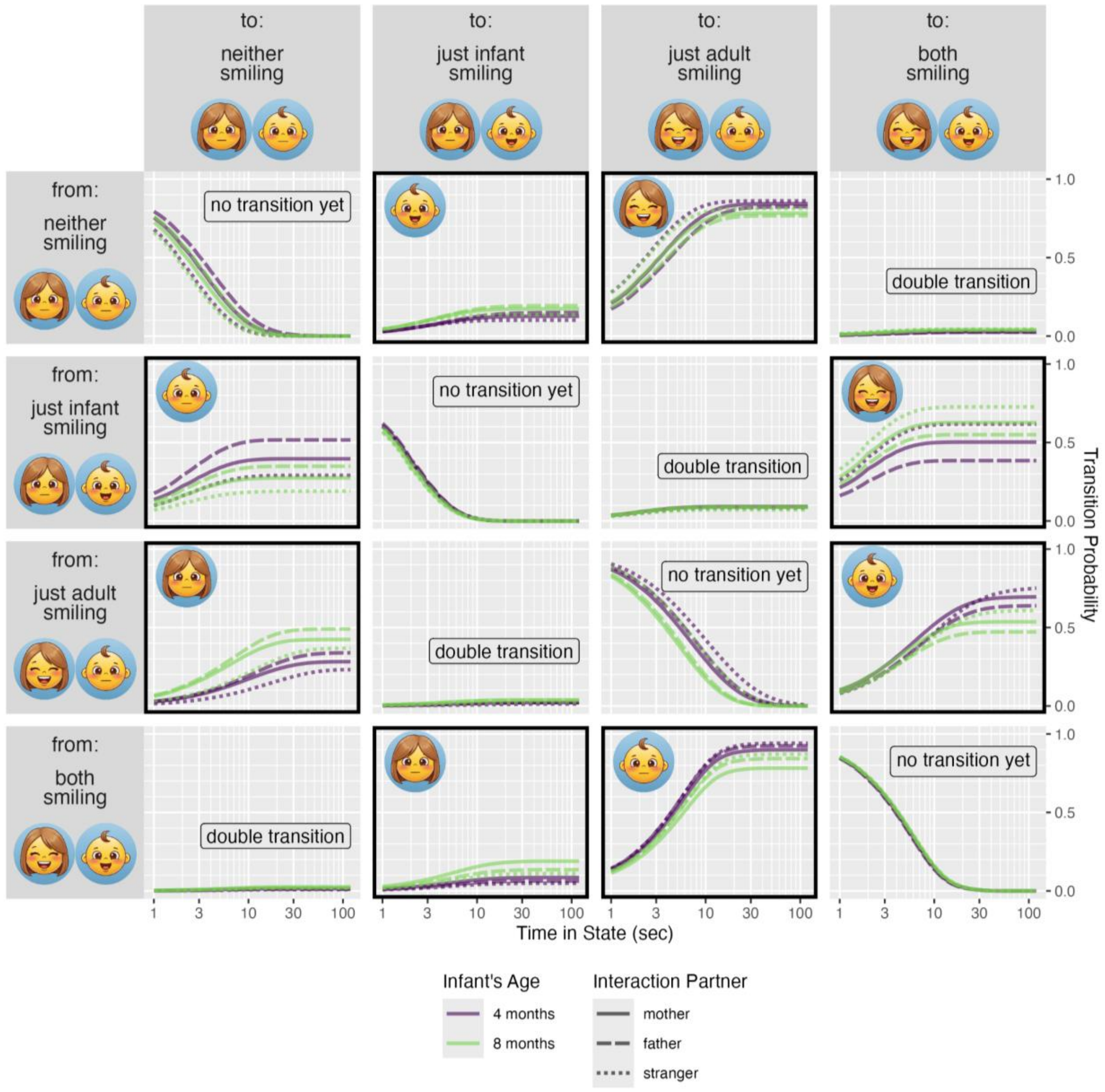

Supplementary Materials G: Infant Gender Effects

Figure S3. Model-averaged survival and cumulative incidence curves for the main effect of Infant Gender. Curves are for other predictors at baseline (i.e., 4-month-olds interacting with their mothers), but these curves are representative of the Infant Gender effect because 2-way and 3-way interactions were not supported by model fit.

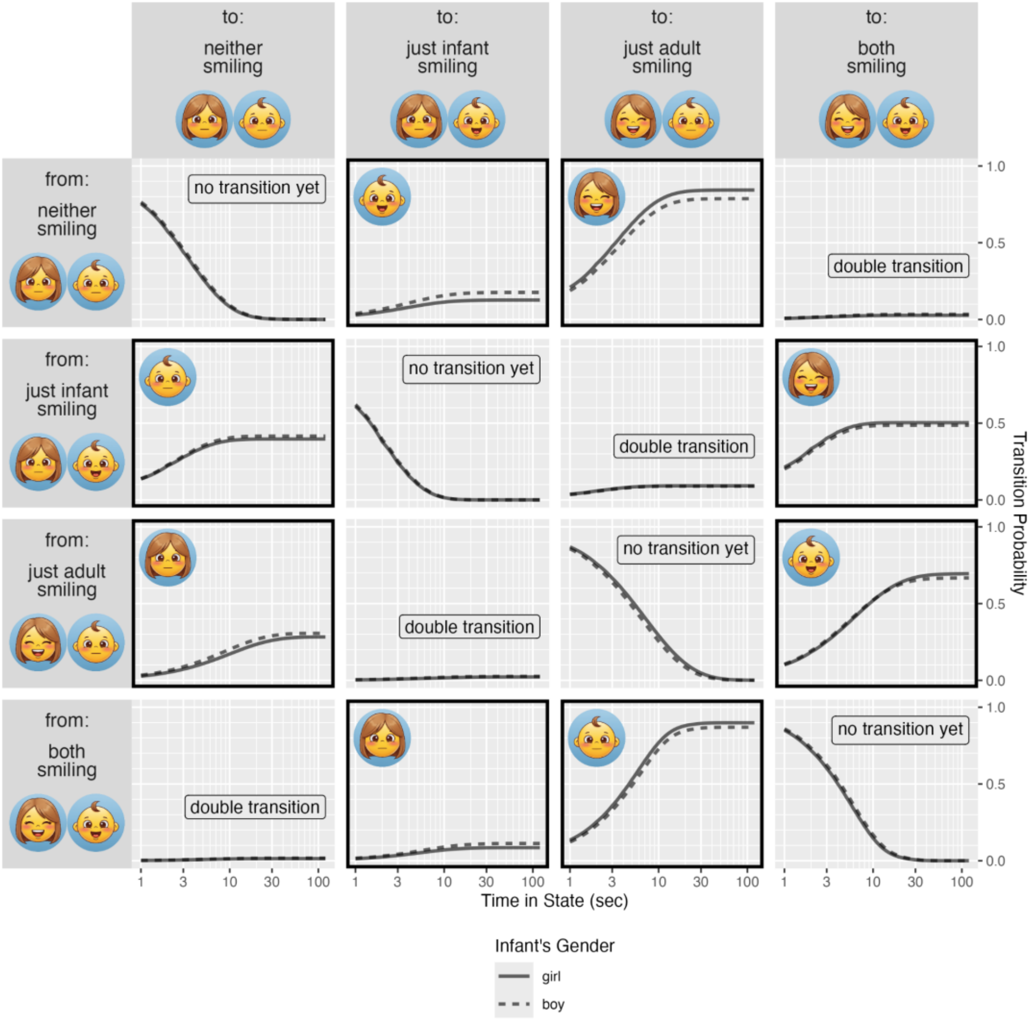

Supplement: Supplementary file 1 [file rstb.2025.0142.zip › rstb.2025.0142/rstb20250142_si_001.pdf]
